# The cross-talk between GSK-3β, RKIP, and PTEN as potential targets for therapeutic implications in cancer: a comprehensive insight

**DOI:** 10.3389/jpps.2026.16610

**Published:** 2026-06-25

**Authors:** Esraa M. Mosalam, Mahmoud S. Abdallah, Ahmed R. Gardouh, Eman Hamza, Mostafa M. Bahaa, Mahmoud Nazih, Reham A. Al-Dhelaan, Noha Kamal

**Affiliations:** 1 Department of PharmD, Faculty of Pharmacy, Jadara University, Irbid, Jordan; 2 Department of Clinical Pharmacy, Faculty of Pharmacy, University of Sadat City (USC), Sadat, Egypt; 3 Department of Pharmaceutical Sciences, Faculty of Pharmacy, Jadara University, Irbid, Jordan; 4 Department of Biochemistry, College of Medicine, Imam Mohammad Ibn Saud Islamic University (IMSIU), Riyadh, Saudi Arabia; 5 Pharmacy Practice Department, Faculty of Pharmacy, Horus University, New Damietta, Egypt; 6 Pharmacy Practice Department, Faculty of Pharmacy, Mansoura National University, Gamasa, Egypt; 7 Department of Clinical Pharmacy, Faculty of Pharmacy, Deraya University, Minya, Egypt; 8 Scientific Office, Egyptian Society of Pharmacogenomics and Personalized Medicine (ESPM), Cairo, Egypt

**Keywords:** cancer mechanisms, GSK-3, GSK-3β, kinases, mTORC1

## Abstract

The serine/threonine kinase glycogen synthase kinase-3 (GSK-3) was initially identified and studied in the regulation of glycogen synthesis. In some cases, suppression of GSK-3 activity by phosphorylation by Akt and other kinases has been associated with cancer progression. In these cases, GSK-3 has tumor suppressor functions. In other cases, GSK-3 has been associated with tumor progression by stabilizing components of the beta-catenin complex. In these situations, GSK-3 has oncogenic properties. The Raf kinase inhibitor protein (RKIP) has been reported to be under expressed in many cancers and plays a role in the regulation of tumor cells’ survival, proliferation, invasion, and metastasis, hence, a tumor suppressor. RKIP also regulates tumor cell resistance to cytotoxic drugs/cells. Likewise, the tumor suppressor, phosphatase and tensin homolog (PTEN), which inhibits the phosphatidylinositol 3 kinase (PI3K)/protein kinase B (Akt) pathway, is either mutated, under expressed, or deleted in many cancers and shares with RKIP its anti-tumor properties and its regulation in resistance. Several pathways are regulated by RKIP, GSK-3, PTEN, and the transcriptional and post-transcriptional regulations of RKIP, GSK-3, and PTEN are significantly altered in cancers. In addition, RKIP, GSK-3 and PTEN play a key role in the regulation of tumor cells response to chemotherapy and immunotherapy. In this review, we will focus on the roles that GSK-3, PTEN, and RKIP play in various human cancers. We will also discuss how this pivotal kinase interacts with multiple signaling pathways such as: PI3K/PTEN/Akt/mechanistic target of rapamycin complex 1 (mTORC1), nuclear Factor kappa-B (NF-κB)/Snail family transcriptional repressor 1 (Snail)/Yin Yang 1 (YY1) loop, and rat sarcoma virus oncogene (Ras)/rapidly accelerated fibrosarcoma (Raf)/mitogen-activated protein kinase (MEK)/extracellular signal-regulated kinase (ERK).

## Introduction

Glycogen synthase kinase 3 beta (GSK-3β), which is a serine/threonine kinase was initially identified because of its key role in the regulation of glycogen synthesis. But it is now well-established that GSK-3 performs critical functions in many cellular processes, such as apoptosis, tumor growth, cell invasion, and metastasis [1]. There is conflicting evidence on the effect of GSK-3 inhibition on cancer cell development. It can act as a tumor promoter as well as a tumor suppressor in different types of cancer [2]. However, it is found that combination of GSK-3β inhibitors with chemotherapeutic agents can significantly reduce the resistance of various tumor cells towards chemotherapy [3]. In the last few years, many GSK-3 inhibitors have been developed, and some are currently being tested in clinical trials [4]. Indazoles are nitrogen-based heterocyclic chemicals that possess many types of biological activities and representatives of this class of pharmacological agents are widely used as antibacterial, anti-inflammatory, anti-HIV, antiprotozoal and antimalarial agents [5]. Recent studies are showing promising activity of indazole derivatives as anticancer agents [6].

The Raf kinase inhibitor protein (RKIP) has been reported to be under expressed in many cancers and acts as a suppressor to the regulation of tumor cells’ survival, proliferation, invasion, and metastasis. RKIP also regulates tumor cell resistance to cytotoxic drugs/cells. Likewise, the tumor suppressor, phosphatase and tensin homolog (PTEN), which inhibits the phosphatidylinositol 3 kinase (PI3K)/Akt pathway, is either mutated, under expressed, or deleted in many cancers and shares with RKIP its anti-tumor properties and its regulation in resistance [7]. The underlying mechanism of the interrelationship between the signaling expressions of RKIP and PTEN in cancer is not clear [7]. RKIP and PTEN play a key role in the regulation of tumor cells response to chemotherapy and immunotherapy [7]. There are crosstalks involving the mitogen-activated protein kinase (MAPK)/PI3K pathways and the dysregulated nuclear factor kappa-light-chain-enhancer of activated B cells (NF-κB)/Snail/Yin Yang 1 (YY1)/RKIP/PTEN loop in many cancers [7].

While the individual biological roles of GSK-3β, RKIP, and PTEN have been extensively characterized, increasing evidence indicates that their cooperative and interconnected signaling functions are particularly relevant in cancer. Rather than acting as isolated regulators, these molecules converge at critical signaling nodes including the PI3K/PTEN/Akt/GSK-3β axis and the NF-κB/Snail/YY1 regulatory loop to jointly influence tumor proliferation, epithelial-mesenchymal transition, metastasis, and resistance to therapy. Accordingly, this review emphasizes the functional crosstalk among GSK-3β, RKIP, and PTEN, highlighting how their coordinated dysregulation reshapes oncogenic signaling networks and presents opportunities for therapeutic targeting.

A comprehensive literature search was conducted to identify studies exploring the cross-talk between GSK-3β, RKIP, and PTEN and their therapeutic implications in cancer Relevant studies were retrieved from PubMed, Scopus, Web of Science, and Embase between 1990 and 2026. They were systematically searched using combinations of controlled vocabulary (MeSH terms) and free-text keywords. Search terms included combinations of “GSK-3β”, “RKIP”, “PTEN”, “PI3K/Akt”, “NF-κB”, “Wnt/β-catenin”, “epithelial–mesenchymal transition”, “drug resistance”, “tumor microenvironment”, and “GSK-3β inhibitors”. Filters included English-language, peer-reviewed original, and review articles. Reference lists of included studies were screened for additional relevant articles. Approximately 400 records were initially identified, of which 263 peer-reviewed articles were selected following screening for relevance, duplication, and scientific quality. Emphasis was placed on mechanistic studies, translational research, preclinical investigations, and clinical studies relevant to the role of GSK-3β and its interaction with RKIP/PTEN signaling in cancer progression, metastasis, immune modulation, and therapeutic resistance. Seminal studies were included to provide historical and mechanistic context, while recent publications were prioritized to reflect current advances in the field.

## Glycogen synthase kinase-3 (GSK-3)

### Isoforms: GSK-3α and GSK-3β

**FIGURE 1:**
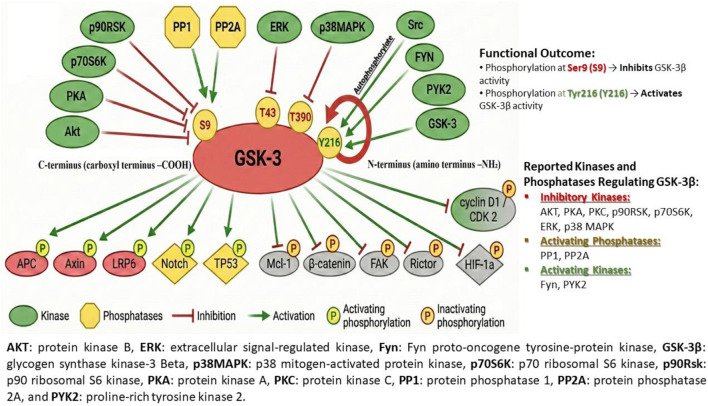
Sites of Phosphorylation of GSK-3β which Regulate its Activity. Schematic representation of the regulatory phosphorylation sites controlling GSK-3β activity. Inhibitory phosphorylation at Ser9 by upstream kinases—including Akt, PKA, p90RSK, and p70S6K—is indicated by inhibitory arrows and results in reduced GSK-3β activity. In contrast, phosphorylation at Tyr216, mediated by kinases such as Fyn and PYK2 or via autophosphorylation, is associated with GSK-3β activation. Protein phosphatases PP1 and PP2A can reverse inhibitory Ser9 phosphorylation and restore kinase activity. This figure highlights how diverse signaling inputs dynamically regulate GSK-3β function under physiological and pathological conditions, including cancer. Akt: protein kinase B, ERK: extracellular signal-regulated kinase, Fyn: Fyn proto-oncogene tyrosine-protein kinase, GSK-3β: glycogen synthase kinase-3 Beta, p38MAPK: p38 mitogen-activated protein kinase, p70S6K: p70 ribosomal S6 kinase, p90Rsk: p90 ribosomal S6 kinase, PKA: protein kinase A, PKC: protein kinase C, PP1: protein phosphatase 1, PP2A: protein phosphatase 2A, and PYK2: proline-rich tyrosine kinase 2.

The GSK-3 gene family contains two highly conserved kinases, GSK-3α and GSK-3β [8]. Both GSK-3α and GSK-3β have strong preferences for primed substrates, which means they prefer substrates that have already been phosphorylated by other kinases [e.g., casein kinase1 (CK1), mitogen-activated protein kinases (MAPK), extracellular regulated Kinase (ERK), p38MAPK, and c-Jun N-terminal kinase (JNK), 5’ adenosine monophosphate-activated protein kinase (AMPK)] and others (Figure 1).

### GSK-3 activity is controlled by phosphorylation/dephosphorylation

**FIGURE 2:**
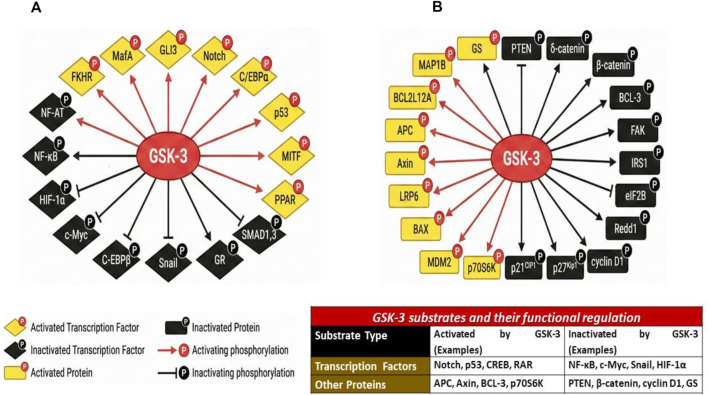
Diversity of GSK-3 Substrates. **(A)** Transcription factors regulated by GSK-3. This panel illustrates transcription factors that are directly phosphorylated by GSK-3 and whose activity is thereby modulated. Transcription factors activated by GSK-3–mediated phosphorylation are depicted as yellow diamond shapes with black lettering, with phosphorylation events indicated by red “P” symbols inside red circles. Transcription factors inactivated by GSK-3 phosphorylation are shown as black diamond shapes with white lettering, with inhibitory phosphorylation indicated by black “P” symbols inside black circles. These regulatory events influence key cellular processes, including proliferation, apoptosis, differentiation, inflammation, and epithelial–mesenchymal transition. **(B)** Non-transcriptional protein substrates of GSK-3. This panel summarizes additional signaling, metabolic, and structural proteins phosphorylated by GSK-3. Proteins whose function or stability is enhanced upon phosphorylation are represented as yellow rectangles with black lettering, whereas proteins whose activity or stability is suppressed by phosphorylation are shown as black rectangles with white lettering. Phosphorylation events are indicated by red or black “P” symbols corresponding to activating or inhibitory outcomes, respectively. In certain cases, the same substrate may be differentially activated or inhibited by GSK-3 depending on cellular context, subcellular localization, or upstream signaling inputs. Together, this figure highlights the breadth of GSK-3 substrate specificity and underscores its role as a central signaling integrator that regulates transcriptional control, cell-cycle progression, apoptosis, metabolism, cytkeletal dynamics, and therapy-response pathways in both physiological and pathological contexts. Akt: protein kinase B, APC: adenomatous polyposis coli, Axin: axis inhibition protein, BAX: Bcl-2-associated X protein, BCL-3: B-cell lymphoma 3, BCL2L12A: Bcl-2-like protein 12A, β catenin: β-catenin (cadherin-associated protein), C/EBPα: CCAAT/enhancer-binding protein alpha, cEBPβ: CCAAT/enhancer-binding protein beta, c-Myc: cellular myelocytomatosis oncogene, CREB: cAMP response element-binding protein, cyclin D1: cyclin D1 protein, eIF2B: eukaryotic translation initiation factor 2B, ERK: extracellular signal-regulated kinase, FAK: focal adhesion kinase, FKHR: forkhead in rhabdomyosarcoma (FOXO1), Fyn: Fyn proto-oncogene tyrosine-protein kinase, GLI3: GLI family zinc finger 3, GR: glucocorticoid receptor, GS: glycogen synthase, GSK-3: glycogen synthase kinase-3, GSK-3β: glycogen synthase kinase-3 Beta, HIF-1 alpha: hypoxia-inducible factor 1-alpha, IRS1: Insulin receptor substrate 1, Jun-B,C,D: Jun proto-oncogene family members B, C, and D, LRP6: low-density lipoprotein receptor-related protein 6, MafA: musculoaponeurotic fibrosarcoma oncogene homolog A, MAP18: microtubule-associated protein 18, MAP2C: microtubule-associated protein 2C, Mcl1: myeloid cell leukemia sequence 1, MDM2: mouse double minute 2 homolog, MLK3: mixed-lineage kinase 3, MTF: metal-responsive transcription factor, NF-AT: nuclear factor of activated T-cells, NF-κB: nuclear factor kappa B, Notch: Notch signaling receptor, Nrf2: nuclear factor erythroid 2–related factor 2, p130Rb: retinoblastoma-like protein 2, p21CIP1: cyclin-dependent kinase inhibitor 1A, p27Kip1: cyclin-dependent kinase inhibitor 1B, p38MAPK: p38 mitogen-activated protein kinase, p53: tumor protein 53, p70S6K: p70 ribosomal S6 kinase, p90Rsk: p90 ribosomal S6 kinase, PKA: protein kinase A, PKC: protein kinase C, PPAR: peroxisome proliferator-activated receptor, PP1: protein phosphatase 1, PP2A: protein phosphatase 2A, PTEN: phosphatase and tensin homolog, PYK2: proline-rich tyrosine kinase 2, RAR: retinoic acid receptor, Redd1: regulated in development and DNA damage response 1, sigma catenin: σ-catenin (Plakoglobin), SMAD 1,3: SMAD family member 1 and 3, Snail: SNAIL family transcriptional repressor 1, and TSC2: tuberous sclerosis complex 2.

GSK-3α and GSK-3β are expressed ubiquitously and highly conserved. The activity of GSK-3α is extinguished by phosphorylation at S21, while GSK-3β activity is silenced by phosphorylation at S9 [9, 10]. Phosphorylation of GSK-3β at S9 leads to its inactivation by proteasomal degradation and has been associated with many pathological conditions, including cancer. Various kinases phosphorylate GSK-3β at S9 including protein kinase A (PKA), protein kinase B (PKB a.k.a Akt), p90 ribosomal S6 kinase (p90Rsk), p70 ribosomal S6 kinase (p70S6K) [10–12]. A diagram depicting sites of regulation of GSK-3β is presented in Figure 2.

### Biochemical functions of GSK-3

GSK-3β represses the expression of certain immediate response genes in quiescent cells [13]. GSK-3 can alter the activity of proteins important in RNA translation such as p70S6K [14]. The GSK-3-related yeast protein mitotic casein kinase 1 (Mck1) can inhibit the activity of the major mitotic cyclin-dependent kinase (Cdk) complex cyclin B2–cyclin-dependent kinase 1 complex (Clb2-Cdk1) and regulate cellular division [15]. A diagram illustrating some of the targets of GSK-3 is presented in Figure 2.

Nitrogen-containing heterocycles serve as a major scaffold for a variety of biological substances and pharmaceuticals [16]. Indazole is one of these chemicals with biological, agricultural, and industrial applications. Anti-inflammatory, anti-tumor, anti-HIV, anti-platelet, and serotonin receptor antagonist properties are all present in indazole and its derivatives [16]. The basic structure of various medicinal compounds, such as Granisetron, a 5HT3 receptor antagonist used as an anti-emetic drug [17], and Benzydamine, an anti-inflammatory medication, is formed from indazole derivatives [18]. Because of the planarity of the indazole ring, side chain length, and fictionalizations at various points, an enormous variety of indazole derivatives can be created, resulting in novel compounds with biological and medicinal capability [16].

Indazole derivatives represent a prominent chemotype among small-molecule GSK-3β inhibitors, owing to their ability to engage the ATP-binding pocket and establish key hinge-region interactions. From a structure–activity perspective, substitution at the indazole core strongly influences kinase potency and selectivity, particularly through modulation of hydrogen-bonding capacity and steric complementarity within the active site [19, 20]. Substituents at the 3- and 5-positions of the indazole ring frequently project into hydrophobic subpockets adjacent to the gatekeeper residue, where increased hydrophobic bulk is associated with enhanced potency but also raises the risk of cross-reactivity with structurally related kinases [6].

Structure–activity relationship analyses further reveal that electron-withdrawing substituents on appended aryl or heteroaryl groups can improve biochemical potency by stabilizing binding orientation, whereas excessive polarity often compromises cellular permeability. Conversely, incorporation of flexible linkers or bulky aromatic extensions may improve cellular activity but reduce kinase selectivity, highlighting a common trade-off between potency and specificity within this chemical class [21]. Selectivity profiling demonstrates that many indazole-based GSK-3β inhibitors exhibit off-target activity against cyclin-dependent kinases and other serine/threonine kinases that share homologous hinge motifs [22].

### Context-dependent determinants of GSK-3β function in cancer

The apparently contradictory tumor-suppressive and oncogenic roles of GSK-3β can be reconciled by considering the strong context dependency of its signaling functions [23]. GSK-3 act as a tumor suppressor as it inhibits of glycogen synthase, which is the rate-limiting kinase in glycogen production that is crucial for cancer cell survival and proliferation [24].

**FIGURE 3:**
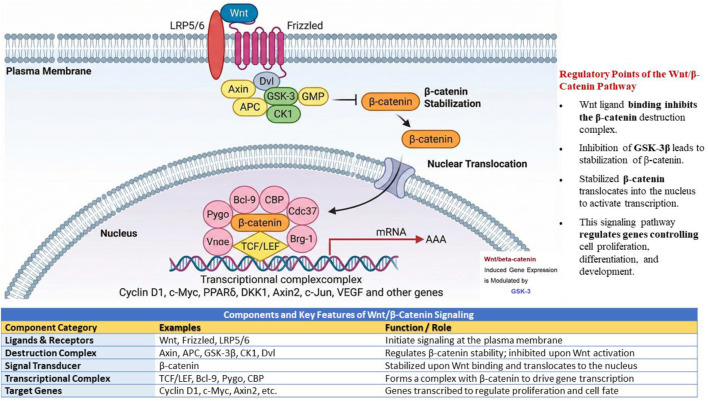
Wnt/beta-catenin Induced Gene Expression is Modulated by GSK-3. Schematic representation of canonical Wnt/β-catenin signaling and its modulation by GSK-3β. In the presence of Wnt ligands, Wnt binds to its cell-surface coreceptors Frizzled (Fz) and LRP5/LRP6, initiating pathway activation. The Frizzled receptor is depicted as a seven-pass transmembrane protein (squiggly line), whereas LRP5/LRP6 are shown as single-pass transmembrane receptors (red ovals). Upon receptor engagement, components of the β-catenin destruction complex—including GSK-3β and casein kinase-1 (CK1)—are functionally inhibited, resulting in stabilization and accumulation of β-catenin in the cytoplasm. Stabilized β-catenin translocates to the nucleus, where it complexes with transcription factors of the TCF/LEF family (represented by yellow diamonds) to activate Wnt-responsive gene transcription. Proteins that interact with receptor-proximal signaling elements or with nuclear transcriptional complexes are indicated by yellow ovals and pink circles, respectively. Directional arrows denote signaling activation, whereas inhibitory interactions within the destruction complex highlight the suppressive role of GSK-3β in the absence of Wnt signaling. This figure illustrates how GSK-3β functions as a central negative regulator of β-catenin stability, thereby controlling transcriptional programs involved in cell proliferation, differentiation, survival, and tumorigenesis, and emphasizes the context-dependent nature of GSK-3β signaling in cancer. AAA: ATPases associated with diverse cellular activities, APC: adenomatous polyposis coli, Axin: axis inhibition protein, Axin2: axis inhibition protein 2, Bcl9: B-cell lymphoma 9, Brg: Brahma-related gene (chromatin remodeling protein), CBP: CREB-binding protein, Cdc37: cell division cycle 37, CK1: casein kinase 1, Cyclin D1: cyclin D1 protein, c-Jun: Jun proto-oncogene, c-Myc: cellular myelocytomatosis oncogene, DKK1: Dickkopf-related protein 1, Dvl: Dishevelled segment polarity protein, GMP: guanosine monophosphate, GSK-3: glycogen synthase kinase-3, LRP5/6: low-density lipoprotein receptor-related protein 5/6, PPAR gamma: peroxisome proliferator-activated receptor gamma, pygo: pygopus transcription coactivator, TCF/LEF: T-cell factor/lymphoid enhancer-binding factor, VEGF: vascular endothelial growth factor.

GSK-3β acts as a signaling hub whose biological output is shaped by cell type, upstream pathway activation, subcellular localization, and tumor stage [25]. In tumors driven by aberrant Wnt/β-catenin signaling, active GSK-3β promotes β-catenin degradation and suppresses proliferation, thereby exerting tumor-suppressive effects [26] (Figure 3). Conversely, in cancers characterized by chronic NF-κB activation or inflammatory signaling, GSK-3β can enhance NF-κB–dependent transcriptional activity, thereby supporting tumor survival and chemoresistance [27].

Upstream regulation by the PI3K/PTEN/Akt pathway further modulates GSK-3β activity, such that loss of PTEN or hyperactivation of Akt leads to inhibitory phosphorylation of GSK-3β at Ser9, altering its downstream targets [28]. In addition, cytoplasmic versus nuclear localization of GSK-3β influences substrate accessibility, determining whether it preferentially regulates oncogenic transcription factors (e.g., NF-κB, Snail) or cell-cycle regulators (e.g., cyclin D1, c-Myc) [29]. Importantly, the role of GSK-3β may also evolve during tumor progression, functioning as a tumor suppressor in early stages while promoting invasion, therapy resistance, or metastatic traits at later stages [30].

Together, these findings indicate that GSK-3β cannot be classified as inherently oncogenic or tumor suppressive; rather, its functional role reflects the integrated oncogenic signaling landscape in which it operates. The therapeutic implications of targeting GSK-3β depend on signaling context, emphasizing that successful clinical translation requires stratification based on pathway dominance rather than indiscriminate kinase inhibition (Table 1).

**TABLE 1:** GSK-3β phosphorylation patterns and oncogenic role.

Cancer type	Functional consequence and references	Main effect of GSK-3β on tumor
Lung cancer	Wnt/β-catenin signaling suppresses GSK-3β, increases free β-catenin, and upregulates E-cadherin, with loss of Wnt7a potentially contributing to tumor formation or progression [31] Inhibition of GSK-3β increases involucrin expression through AP-1 suppression, promotes squamous differentiation [30]	Tumor-promoter
Renal cell carcinoma (RCC)	In 9-ING-41, a maleimide-based ATP-competitive GSK-3β inhibitor, induces cell cycle arrest and death, with enhanced anticancer effects [32]	Tumor-promoter
Glioblastoma	GSK-3β promotes glioma stem cell self-renewal; tideglusib disrupts GSK-3β/USP22/KDM1A axis, sensitizing intracranial GBM xenografts to TMZ [33] GSK-3β inhibitor 9-ING-41 enhanced the anticancer efficacy of lomustine in patient-derived xenograft GBM models [34, 35]	Tumor-promoter
Neuroblastoma	Inhibiting GSK-3 with LY2090314 suppresses NB growth [36]	Tumor-promoter
Hepatocellular cancer	Akt phosphorylates GSK-3β in HCC, stimulating tumorigenesis; pGSK-3β correlates with tumor stage and vascular invasion; NF-κB activated via IKK interaction; PDCD6 promotes HCC via Akt/GSK-3β axis [3]. Functional overexpression of GSK-3β also contributes to resistance against therapies such as retinoids and sorafenib [37]. Inhibition of GSK-3β with tideglusib suppresses growth and restores RARβ signaling, thereby enhancing the antitumor activity of sorafenib [38, 39]	Tumor-promoter
Pancreatic cancer	GSK-3β enhances NF-κB activity, promoting proliferation and resistance to apoptosis, [40]. pSer9-GSK-3β promotes β-catenin nuclear translocation; activates cyclin D1 and AP-1; poor prognosis marker in pancreatic cancer; Akt-mediated GSK-3β inactivation central to tumor progression [41, 42] However, GSK-3β also exhibits a tumor-suppressive role by negatively regulating EMT, increasing snail and Zeb1 [43, 44]	Dual and context-dependent role
Prostate cancer	GSK-3β promotes AR transcription paradoxically pro-tumorigenic in PTEN-loss context [41, 45] PI3K inhibition and mutated PTEN stimulate GSK 3β-mediated degradation of β-catenin by inhibiting the PI3K/Akt pathway, thereby hindering the progression of prostate tumors [46, 47]	Dual and context-dependent role
Breast cancer	GSK-3β inactivation promotes migration of breast cancer cells; elevated in HER2+ and basal subtypes [48] GSK-3 knockdown significantly reduced breast cancer cell growth [49]. GSK-3 inhibitors 9-ING-41 and 9-ING-87 selectively decreased the viability of breast, cancer cells [35]. 9-ING-41 enhanced the anticancer activity of irinotecan both *in vitro* and in chemotherapy-resistant patient-derived xenograft model [7, 50] Kinase-dead GSK-3β increased resistance to doxorubicin and tamoxifen and promoted clonogenic growth, while wild-type or constitutively active GSK-3β was less permissive [51]	Dual and context-dependent role
Ovarian cancer	The Wnt/GSK-3 signaling pathway has been implicated in ovarian cancer progression, [52, 53]. The overexpression of GSK-3β increased proliferation through modulation of cell cycle progression and cyclin D1 expression [53]. Elevated cyclin D1 expression, upregulated GSK-3 is associated with chemotherapy resistance [54] Suppression of GSK-3β reduces carboplatin-induced apoptosis [55]	Dual and context-dependent role

Akt: protein kinase B (AKT, serine/threonine kinase), AP-1: activator protein 1, AR: androgen receptor, ATP: adenosine triphosphate, EMT: epithelial–mesenchymal transition, GBM: glioblastoma, GSK-3β: glycogen synthase kinase-3, beta, HER2+: human epidermal growth factor receptor 2 positive, HCC: hepatocellular carcinoma, IKK: IκB kinase, KDM1A: lysine-specific demethylase 1A, NB: neuroblastoma, NF-κB: nuclear factor kappa-light-chain-enhancer of activated B cells, PDCD6: programmed cell death 6, PI3K: phosphoinositide 3-kinase, PTEN: phosphatase and tensin homolog, RCC: renal cell carcinoma, RARβ: retinoic acid receptor beta, TMZ: temozolomide, USP22: ubiquitin-specific peptidase 22, Wnt: wingless-related integration site signaling pathway, Zeb1: zinc finger E-box-binding homeobox 1.

#### GSK-3 as a tumor-promoter

GSK-3 may increase cell proliferation while simultaneously acting as a tumor promoter. In a range of tumor types, including colon, liver, ovarian, and pancreatic malignancies, GSK-3 is overexpressed in the body [45]. When GSK-3β expression was repressed, pancreatic cancer development and angiogenesis were reduced. GSK-3β knocked-down cells have decreased amounts of Bcl-2 and vascular endothelial growth factor (VEGF). GSK-3 inhibitors may be effective in the treatment of tumors where in which GSK-3 is overexpressed [56].

In lung cancer cells, Wnt/β-catenin signaling suppresses GSK-3β, increases free β-catenin, and upregulates E-cadherin, with loss of Wnt7a potentially contributing to tumor formation or progression [31]. Studies in lung cancer models show that cigarette smoke components inhibit GSK-3β, similar to inhibitors as lithium and SB216763, leading to increased involucrin expression via AP-1 suppression and promoting squamous differentiation, highlighting GSK-3β’s role in lung carcinogenesis [30].

In renal cell carcinoma (RCC), 9-ING-41, a maleimide-based ATP-competitive GSK-3β inhibitor, induces cell cycle arrest and death, with enhanced anticancer effects when combined with autophagy inhibitors, cytokine-activated immune cells, or targeted therapies, suggesting a tumor-promoting role of GSK-3β in RCC progression and survival [32].

GSK-3β expression and activity in giloblastoma multiforme (GBM) cell lines were shown to be greater than in normal brain tissue. In 57 human tumor specimens, the active form of GSK-3β (pGSK3y216) was linked to poor progression-free survival and overall survival (OS), indicating a tumor-promoting role and its value as an independent prognostic marker [57]. Inhibition of GSK-3β induces cytotoxicity in c-Myc–active glioma cells and may selectively target GBM stem cell-like populations, supporting its potential as a therapeutic target [57]. Furthermore, the GSK-3β inhibitor 9-ING-41 enhanced the anticancer efficacy of lomustine in patient-derived xenograft GBM models, resulting in significant survival improvement and histologically confirmed cures in treatment-resistant tumors [34, 35].

Neuroblastoma (NB)’s distinct appearance and tumor biology provide difficulties in developing successful treatment regimens, particularly in patients identified as high risk (high v-myc avian myelocytomatosis viral oncogene neuroblastoma derived homolog (MYCN) expression) [58]. In recent years, studies investigating GSK-3 inhibitors in cancer treatment have mostly focused on three compounds: Tideglusib, AR-A011418, and LY2090314 [59]. The data show that inhibiting GSK-3 with LY2090314 suppresses NB growth. In comparison to Tideglusib, low doses of LY2090314 greatly inhibited NB cellular development. This research shows that LY2090314 has the potential to be a future treatment for NB [36]. This indicates a tumor-promoting role of GSK-3 in NB progression and supporting GSK-3 inhibition as a promising therapeutic strategy.

GSK-3β is overexpressed in hepatocellular carcinoma (HCC), where it promotes tumor cell proliferation, colony formation, tumor development, and resistance to apoptosis, indicating a clear tumor-promoting role in HCC progression [38, 60]. Functional overexpression of GSK-3β also contributes to resistance against therapies such as retinoids and sorafenib, partly because sorafenib itself can activate GSK-3β within tumor cells and the tumor microenvironment [37]. Pharmacological inhibition of GSK-3β with tideglusib suppresses HCC xenograft growth and restores RARβ signaling, thereby enhancing the antitumor activity of sorafenib and sensitizing tumors to therapy [38, 39].

#### Dual and context-dependent role of GSK-3β

In colon cancer, there is a dual and context-dependent role of GSK-3β as both a tumor promoter and tumor suppressor. Pharmacological inhibitors of GSK-3β activity and RNA interference inhibit GSK-3β expression causing apoptosis and decreased proliferation in colon cancer cells, suggesting a tumor-promoting role of active GSK-3β in supporting cancer cell survival [61]. In contrast, GSK-3β also exhibits a tumor-suppressive function by negatively regulating β-catenin/TCF signaling, as prostaglandin E2 trans-activated the β-catenin/TCF-dependent activation of TCF-4 in colon cancer cells by inhibiting GSK-3β [62].The activation of TNF-related apoptosis-inducing ligand (TRAIL) by wortmannin was fully abolished when GSK-3, a downstream target of active Akt, was inhibited [63]. In colon cancer cells, PI3K inhibition induces TRAIL production, while GSK-3β inactivation regulates cell division cycle 25 homolog A (Cdc25A) ubiquitin-mediated proteolysis, with GSK-3β suppression during G1 linked to Cdc25A overexpression, elucidating the PI3K/AKT/GSK-3 pathway’s role in intestinal cell homeostasis [64]. The PI3K/Akt pathway inhibits both GSK-3β and checkpoint kinase 1 (CHK1), which promotes cell cycle progression by increasing Cdc25A levels.

In pancreatic ductal adenocarcinoma (PDAC), GSK-3β demonstrates a dual and context-dependent role in tumor progression. Upregulation of GSK-3β enhances NF-κB activity, promoting proliferation, pro-tumorigenic cytokine production, and resistance to apoptosis, supporting a tumor-promoting role in PDAC growth and survival [40]. Consistently, GSK-3β inhibition suppresses NF-κB activation and reduces tumor growth [40]. However, GSK-3β also exhibits a tumor-suppressive role by negatively regulating epithelial–mesenchymal transition (EMT), as its inhibition increases EMT-associated transcription factors such as Snail and Zeb1, thereby promoting invasion and metastasis [43, 44]. These findings highlight the paradoxical functions of GSK-3β in PDAC, suggesting that inhibition of GSK-3β alone may produce unintended pro-metastatic effects. Therefore, combination strategies integrating GSK-3 inhibitors with other targeted therapies may provide a more effective therapeutic approach [40].

In prostate cancer cells, the activity of GSK-3β is essential for androgen-stimulated gene expression. The crosstalk between the PI3K/Akt and androgen pathways is mediated by β-catenin [65]. In prostate cancer cells, GSK-3β is phosphorylated and inactivated by PI3K/Akt signaling, increasing nuclear β-catenin and androgen receptor activity to promote growth and survival, while GSK-3β inhibition sensitizes cells to TRAIL-induced apoptosis via caspase-8, independent of NF-κB activation, suggesting a tumor-promoting role of active GSK-3β in mediating apoptosis resistance [66]. However, other studies showed that PI3K inhibitor LY294002 and tumor suppressor PTEN deleted on chromosome 10 stimulate GSK 3β-mediated degradation of β-catenin by inhibiting the PI3K/Akt pathway, thereby hindering the progression of prostate tumors [46, 47]. This means that GSK-3β has a context-dependent role in prostate cancer because it functions within interconnected signaling networks, particularly the Wnt/β-catenin and androgen receptor (AR) pathways, whose regulation differs from other cancers.

Traditional chemotherapeutic treatments have had little influence on the progression of metastatic breast cancer [67]. In human breast cancer, patients with the highest GSK-3 levels exhibit significantly increased risks of distant relapse, suggesting a tumor-promoting role of GSK-3 in disease progression. Consistently, GSK-3 knockdown significantly reduced breast cancer cell growth, although its antiproliferative effects varied among different cell lines, highlighting tumor heterogeneity and context-dependent responses [49]. Furthermore, the ATP-competitive GSK-3 inhibitors 9-ING-41 and 9-ING-87 selectively decreased the viability of breast, pancreatic, and ovarian cancer cells with minimal toxicity toward non-tumorigenic cells [35]. In breast cancer models, 9-ING-41 enhanced the anticancer activity of irinotecan (CPT-11) both *in vitro* and in chemotherapy-resistant patient-derived xenograft models, indicating that GSK-3 inhibition may help overcome chemoresistance in metastatic breast cancer [7, 50].

GSK-3 also functions as a tumor suppressor in breast cancer. GSK-3 can suppress the Wnt/beta-catenin pathway by phosphorylating beta-catenin which results in the ubiquitin/proteasome-dependent degradation of beta-catenin. Introduction of kinase-inactive (KI) GSK-3β mutants which presumably functioned as inhibitors of the endogenous wild-type (WT) GSK-3β protein stimulated Wnt signaling and mammary tumorigenesis [68]. Enforced expression of kinase-dead (KD) GSK-3β which presumably functioned as inhibitors of the WT GSK-3β protein, promoted tumorigenesis of breast and skin tumors [69]. Overexpression of constitutively-active GSK-3β mutants, in some studies, increased chemosensitivity, cell cycle arrest, and reduced tumorigenicity of breast cancers [70]. Pharmacological inhibition of GSK-3 induced EMT and invasion in breast cancer [43]. In Michigan cancer foundation-7 (MCF-7) breast cancer cells, kinase-dead GSK-3β increased resistance to doxorubicin and tamoxifen and promoted clonogenic growth, while wild-type or constitutively active GSK-3β was less permissive [51]. This paper is one of the clearest examples of GSK3β behaving as a context-dependent regulator of drug response rather than a simple oncogene or tumor suppressor.

The Wnt/GSK-3 signaling pathway has been implicated in ovarian cancer progression, particularly in serous and advanced-stage tumors where GSK-3β is overexpressed, suggesting a tumor-promoting role in ovarian carcinogenesis [52, 53]. The overexpression of GSK-3β increased proliferation of ovarian cancer cell lines, perhaps through modulation of cell cycle progression and cyclin D1 expression [53]. In ovarian cancer, active GSK-3 may promote cell proliferation by phosphorylating and inhibiting glycogen synthase, limiting glycogen synthesis, and increasing glucose metabolism to meet the high energetic demands of rapidly proliferating tumor cells, though its role via NF-κB–mediated transcription remains unclear [54].Resistance to chemotherapy in ovarian cancer can happen through a variety of mechanisms, including activation of the nuclear factor NF-κB, which can lead to diminished cell death and drug resistance [71]. Furthermore, elevated cyclin D1 expression, upregulated GSK-3 in ovarian cancer cells, is associated with chemotherapy resistance [54]. GSK-3β inhibitors, either alone or in combination with other medications, may slow the progression of serous ovarian tumors [72]. In contrast, suppression of GSK-3β enhances proliferation and reduces carboplatin-induced apoptosis in epithelial ovarian cancer cells, suggesting that GSK-3β silencing contributes to chemoresistance and highlighting the potential importance of GSK-3β expression and methylation status as targets for developing genome-guided therapeutic strategies to improve carboplatin chemosensitivity [55].That makes GSK3β look necessary for chemosensitivity in this particular model, which is the opposite of the pro-survival framing in the proliferation papers and reinforces the context dependence.

#### GSK-3β inhibitors with quantitative potency, clinical-stage data and side effects

Several GSK-3β inhibitors with distinct mechanisms and developmental status have been reported. LY2090314, an ATP-competitive inhibitor, exhibits sub-nanomolar potency against GSK-3β (IC_50_ ≈ 0.9 nM) and has entered early-phase clinical evaluation in advanced solid tumors and hematologic malignancies, although its clinical development has been limited by toxicity and narrow therapeutic window [73]. In contrast, 9-ING-41 (elraglusib), a maleimide-based ATP-competitive inhibitor, demonstrates biochemical IC_50_ values in the low-micromolar range (≈0.7 µM for GSK-3β) [74] and has progressed into phase I/II clinical trials for refractory solid tumors and hematologic cancers, both as monotherapy and in combination with chemotherapy [75]. Another clinically explored compound, tideglusib (NP031112), is an irreversible non-ATP-competitive GSK-3β inhibitor with reported enzyme IC_50_ values in the low-nanomolar range (≈5–60 nM depending on assay conditions), originally developed for neurodegenerative diseases and subsequently evaluated in preclinical oncology models [76].

GSK-3β is widely involved in normal physiological processes, including metabolism, neuronal function, and immune regulation; therefore, its therapeutic targeting requires careful safety evaluation. Preclinical studies indicate that toxicities associated with GSK-3β inhibition are generally dose-dependent and reversible, suggesting a therapeutic window with appropriate dosing strategies.

Early-phase clinical trials of selective GSK-3β inhibitors, elraglusib (9-ING-41), have demonstrated a favorable safety profile, with predominantly low-grade and manageable adverse events such as fatigue, gastrointestinal symptoms, and transient visual disturbances. Severe organ-specific toxicities have not been major dose-limiting factors, supporting the feasibility of clinical application [75].

However, given the context-dependent dual role of GSK-3β, excessive or non-selective inhibition may disrupt normal signaling or stabilize oncogenic substrates. Therefore, biomarker-guided patient selection, optimized dosing, and combination strategies are essential to maximize efficacy while minimizing toxicity.

## RKIP and PTEN as coordinated modulators of oncogenic signaling

Most cancers express low levels of the tumor suppressors RKIP and PTEN deleted on chromosome 10 or mutated [77, 78]. The expression of these gene products in various cancers has been reported to inhibit cell proliferation and cell survival, inhibit metastases, and respond to cytotoxic/apoptotic stimuli [79].

It has been reported that many cancers exhibit a dysregulated NF-κB/Snail/YY1/RKIP/PTEN loop that primarily is responsible for the phenotypic properties of cancer cells [80]. In this loop, the overexpression and activities of NF-κB, Snail, and YY1 regulate the inhibition of RKIP and PTEN expressions. In contrast, the inhibition of either of these gene products, NF-κB, Snail, or YY1 resulted in the upregulation of the expressions and activities of both RKIP and PTEN [81–83]. Further, Snail is a transcriptional repressor of RKIP [84] and YY1 is a transcriptional activator of Snail [85] and a repressor of PTEN [86]. In the dysregulated loop, either one of the gene products, directly or indirectly, regulates other gene products in the loop. Thus, RKIP and PTEN regulate each other indirectly. It was hypothesized that, in addition to the indirect regulation between RKIP and PTEN, there may exist a direct regulation via crosstalk signaling pathways [7].

### RKIP characteristics

#### General properties

The role of RKIP was to regulate the Raf-MEK- ERK signaling pathway by binding to the Raf-1 isoform and interfering with MEK phosphorylation [87]. RKIP has been reported to be a metastasis suppressor gene product and also regulates tumor cell resistance to both chemotherapy and immunotherapy [88]. In addition, RKIP was also reported to inhibit EMT [89]. The process of metastasis involves tumor cells disseminating from the primary tumor, passing through the basement membrane, persevering in the circulatory system, and invading the secondary site [90]. RKIP’s role as a metastasis suppressor was initially investigated by Fu et al. comparing the metastatic prostate cancer cell line (c4-2B) to the non-metastatic cell line (LNCaP) [91]. Immunohistochemistry (IHC) results showed that RKIP expression was associated with the suppression of prostate cancer metastasis [91].

#### RKIP expression in cancer

Low levels of RKIP are associated with a high incidence of tumor growth and metastasis in cancer patients [92]. It is important to determine whether RKIP is able to support late metastatic events such as colonization and growth at a distant site. A marked decrease in bone metastasis was observed in breast cancer patients with low RKIP expression [93].

Multiple studies have shown that a loss or reduction in RKIP expression is frequently found in many solid tumor cancers including breast, melanoma, and prostate [94]. In human breast cancer, RKIP must be downregulated for metastasis to develop [95]. Patients with breast cancer exhibited larger-sized tumors and a higher tumor grade when RKIP was lost or reduced [96]. Additionally, RKIP mRNA levels were significantly lower in metastatic breast cancer patients compared to those that were non-metastatic [96]. Similarly, RKIP staining in the melanoma samples exhibited an overall decrease compared to benign lesions [96] (Table 2).

**TABLE 2:** RKIP expression trends across major cancers.

Cancer type	RKIP expression versus normal	Metastatic status	Key associations and references
Prostate cancer	Downregulated	Decrease metastasis	RKIP loss confers metastatic phenotype without affecting primary tumor growth; snail transcriptionally represses RKIP via proximal E-box; decreased expression in metastases versus primary tumors confirmed by IHC in 134 patients [84, 91, 97, 98]
Breast cancer	Downregulated	Absent in distant metastasis	RKIP silencing mediated by EZH2/PRC2 via H3K27 trimethylation; RKIP re-expression suppresses intravasation and bone [93, 99, 100]
Colorectal cancer (CRC)	Downregulated	Reduced in metastasis	RKIP loss independently predicts poor overall survival; meta-analysis of 19 studies (∼3,700 patients) confirms unfavorable OS (HR 0.55, 95% CI 0.46–0.65) and DFS (HR 0.46, 95% CI 0.30–0.62) in digestive-tract cancers including CRC [101, 102]
Gastric cancer	Downregulated	Lost in ∼56% of primary tumors and in ∼90% of lymph node metastasis	RKIP is lost in 56% primary tumors versus 83% expression in normal mucosa; absent in ∼90% of LN metastases; methylation-dependent silencing in gastric cardia adenocarcinoma (62.1% methylation frequency); independent prognostic marker by multivariate analysis [103, 104]
Lung cancer	Downregulated	Lower with higher stage	RKIP downregulated at both mRNA and protein levels; lower expression associated with higher TNM stage and LN metastasis; YY1 and RKIP co-expression analyses confirm diagnostic and prognostic significance [83]
Melanoma	Downregulated	Stronger down-regulation or complete loss in metastasis	Reduced RKIP expression associated with increased RAS/ERK signaling in melanoma cell lines; RKIP loss sustains ERK after BRAF inhibition (bypass mechanism) [105]
Multiple myeloma	Overexpression of inactive phosphorylated RKIP	Higher ratio of RKIP/p-RKIP correlated with poor progression and a low ratio correlated with progression	RKIP inhibits NF-κB independently of RAF/MEK in MM; phosphorylated (inactive) RKIP (p-RKIP) overexpressed versus active RKIP; high p-RKIP:RKIP ratio correlates with bortezomib resistance; dephosphorylation with PKC inhibitor restores bortezomib sensitivity [106, 107]

BRAF: B-Raf proto-oncogene serine/threonine kinase, CI: confidence interval, CRC: colorectal cancer, DFS: disease-free survival, ERK: extracellular signal-regulated kinase, EZH2: enhancer of zeste homolog 2, H3K27: histone H3 lysine 27, HR: hazard ratio, IHC: immunohistochemistry, LN: lymph node, MEK: mitogen-activated protein kinase, MM: multiple myeloma, mRNA: messenger ribonucleic acid, NF-κB: nuclear factor kappa-light-chain-enhancer of activated B cells, OS: overall survival, p-RKIP: phosphorylated Raf kinase inhibitory protein, PKC: protein kinase C, PRC2: polycomb repressive complex 2, RAF: rapidly accelerated fibrosarcoma kinase, RAS: rat sarcoma viral oncogene homolog, RKIP: raf kinase inhibitory protein, TNM: tumor-node-metastasis, YY1: Yin Yang 1 transcription factor.

#### RKIP-mediated signaling

The Raf-MEK-ERK signaling pathway plays a critical role in the control of cell proliferation, differentiation, migration, and apoptosis. The pathway begins when the activated receptor tyrosine kinases (RTKs) bind with the guanine nucleotide exchange factor, son of sevenless (SOS). Afterward, Ras becomes activated as GDP gets replaced for GTP, which activates Raf. Active Raf phosphorylates MEK, which activates ERK. The attachment of RKIP to Raf inhibits the phosphorylation of MEK. This, in turn, negatively regulates the flow of signals down the Raf-MEK, ERK pathway [108]. RKIP is also able to bind to MEK, and to a lesser extent ERK. When RKIP binds to the kinase domain of Raf-1, it prevents its phosphorylation by PAK and Src kinases at Ser338 and Tyr340/341 [109]. By blocking the Raf-1-MEK-ERK signaling cascade, RKIP inhibits downstream the AP1 transcription factor. Through the modulation of the MAPK pathway, RKIP encourages a balanced cell cycle kinetics and replication process through differential regulation of various pathways including cell proliferation and apoptosis [110, 111]. Upregulation of RKIP shortened the nuclear envelop breakdown (NEB) to anaphase time, and the downregulation of RKIP accelerates the time from NEB to anaphase [96]. Furthermore, RKIP depletion induces the expression of NEK6 (a molecule known to enhance G2/M transition) while simultaneously down-regulating G2/M checkpoint molecules like Aurora B, cyclin G1, and sirtuin [111]. Their results suggested that subtle changes in cell cycle kinetics may be fundamental to RKIP’s role as a metastasis suppressor [111].

**FIGURE 4:**
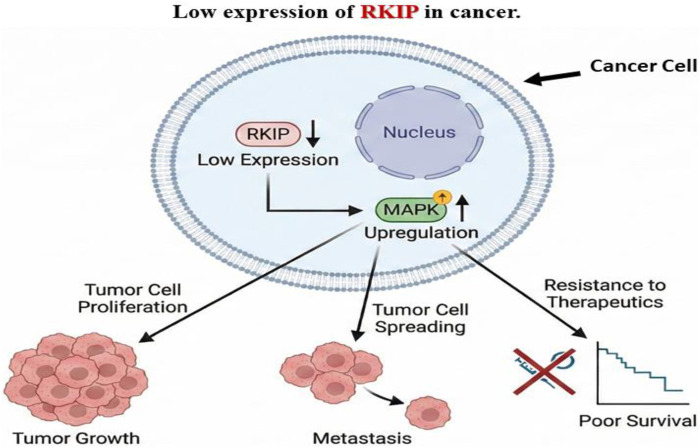
Low expression of RKIP in cancer. Schematic diagram illustrating the biological consequences of downregulation or loss of Raf kinase inhibitor protein (RKIP) in cancer cells. Under physiological conditions, RKIP acts as a negative regulator of the Raf-1/MEK/ERK (MAPK) signaling pathway by inhibiting Raf-1 activity. Loss or reduced expression of RKIP releases this inhibitory constraint, resulting in constitutive activation of the MAPK pathway, as indicated by directional arrows. Sustained MAPK signaling drives multiple oncogenic processes, including enhanced tumor cell proliferation, increased migratory and invasive capacity, and resistance to cytotoxic and targeted therapies. These signaling alterations collectively promote tumor growth, metastatic dissemination, therapy resistance, and poor clinical outcomes. This figure highlights RKIP’s function as a metastasis and resistance suppressor and underscores how its loss shifts signaling balance toward aggressive, treatment-refractory tumor phenotypes. MAPK: mitogen-activated protein kinase, Raf: rapidly accelerated fibrosarcoma, RKIP: Raf kinase inhibitor protein.

Furthermore, RKIP inhibits NF-κB activation by blocking the inhibitor of kappa B (IκB) phosphorylation by a family of IκB kinase (IKK) enzymes. The binding of RKIP to all IKK kinases, including IKK, include NF-κB-inducing kinase (NIK), and transforming growth factor B-activated kinase-1 (TAK-1), inhibits the activation of the NF-κB cascade. RKIP can become phosphorylated by protein kinase C (PKC), which will release the binding of Raf-1 and the subsequent activation of MEK and ERK (Figure 4) [112].

#### Regulation of RKIP expression

##### Transcriptional regulation of RKIP expression

The downregulation of RKIP expression in multiple types of human cancers is also a result of decreased RKIP transcription. A luciferase assay used on human melanoma A375 and cervical cancer HeLa cells revealed that the full RKIP promoter activity requires the nucleotide region compromising −56 to +261 relative to the TSS [113]. The three kinds of cis-acting elements and transcription factors that showed to positively regulate RKIP transcription were specificity protein 1 (Sp1), cyclic adenosine monophosphate (cAMP) response CREB, and the p300 acetylase protein [113].

The knockdown of Sp1 I and second oligonucleotide (Sp1 II) or mutation of these elements decreased RKIP promoter activity [113]. Similarly, the interaction between CREB and transcription factor II B (TFIIB) and TFIID enhances RKIP transcription [113]. The mutated and deleted CREB binding sites resulted in ∼60% reduction in luciferase activity. Thus, it was concluded that CREB has a positive correlation to RKIP promoter activity [113].

The acetyltransferase p300 increases the rate of RKIP transcription by decondensing the tightly packed chromatin [113]. A study indicated that p300 is one of the major transcription factors that promote RKIP transcription [113]. Androgen receptors (ARs) are another type of transcription factor that directly binds to and regulates RKIP transcription in prostate epithelial cells [114]. Dihydrotestosterone (DHT) activates AR, thereby leading to the positive regulation of RKIP [114, 115].

One type of transcription factor that inhibits RKIP expression is Snail. Snail is a zinc finger transcriptional repressor that operates by binding to the E-box cis-elements in the RKIP promoter and recruiting mSin3A histone deacetylases containing repressor complexes [84]. Enhancer of zeste homolog 2 (EZH2) negatively regulated RKIP transcription [82]. EZH2 accelerates cancer cell invasion from RKIP inhibition, but this is dependent on the recruitment of Snail to the RKIP promoter [82]. When Snail is present, EZH2 inhibits RKIP expression at the transcriptional level, which accelerates cellular invasion [106].

Another type of RKIP transcription repressor is the BTB and CNC homology protein 1 (BACH1). BACH1 is a basic leucine zipper transcription factor that was found to enhance the malignancy of breast cancer cells when expression levels were high [116]. The relationship between BACH1 and RKIP exemplifies a double-negative (overall positive) feedback loop by mutually repressing each other’s expression [117]. RKIP suppressed BACH1 expression indirectly through signaling, transcriptional, and RNA interference (let-7) pathways while BACH1 negatively regulated RKIP expression [117]. BACH1 mRNA is a direct let-7 target, and RKIP regulates BACH1 via let-7 binding [118].

##### Epigenetic regulation of RKIP expression

One type of molecule that plays an important role in the methylation of RKIP histones is the epigenetic silencer EZH2. EZH2 is the catalytic subunit of the multicomponent protein complex called polycomb repressive complex 2 (PRC2) and is important in epigenetics as it affects the initiation and progression of several diseases [119]. EZH2 functions by binding to the proximal E-box of the RKIP promoter, recruiting the suppressor of zeste 12 (Suz12), and inducing the tri-methylation of lysines 9 and 27 of histone 3 (H3-K27) [115]. The methylation of lysine amino acids on histones results in reduced transcription, and expression levels between RKIP and EZH2 were shown to have a negative correlation in breast and prostate cell lines as well as in clinical tissues [82].

##### Post-transcriptional regulation of RKIP expression

Several microRNAs (miRNAs) have been identified as important post-transcriptional regulators for RKIP expression [120]. They function by binding to the 3′ UTR of their target mRNAs, inhibiting their translation and the production of a mature protein. One type of miRNA that was identified to suppress RKIP expression is miR-27a. The upregulation of miR-27a decreased RKIP expression in cisplatin-resistant lung adenocarcinoma (LUAD) cell lines [121]. The downregulation of RKIP occurred at the protein rather than mRNA level, indicating probable post-translational regulation [122]. Overexpression of miR-23a was also found to decrease RKIP mRNA and protein expressions [121]. RKIP expression is mediated via direct binding to the miR-23a region [123]. Similarly, overexpression of miR-543 was found to downregulate RKIP expression in clinical prostate cancer specimens and promote the proliferation and metastasis of cancer cells [124]. Moreover, miR-224 expression was significantly upregulated in breast cancer cell lines and enhanced metastasis [125].

##### Post-translational regulation of RKIP expression

The addition of a phosphate group to a mature RKIP protein post-translationally alters RKIP’s conformation as well as its function [112]. The phosphorylation of RKIP of Ser153 inactivates the binding pocket of RKIP, thereby resulting in the release of Raf-1 and activation of MEK and ERK [112]. pRKIP has a higher affinity to GRK2, which normally acts as a negative regulator of the G protein coupled receptor (GPCR) [115]. The pRKIP dimerizes GRK2 and prevents it from inhibiting the GPCR cascade, resulting in enhanced MAPK signaling [92].

### Characteristics of PTEN

#### General properties

PTEN was independently identified in 1997 by three research groups following observations of frequent loss of heterozygosity on the long arm (q arm) of chromosome 10 in glioblastoma, suggesting the presence of a critical tumor suppressor gene in this region. [126–131]. Chromosome 10 possesses a mutated gene that is present in glioblastoma multiforme [132]. Studies found that PTEN was mutated not only in glioblastoma multiforme, but also prostate carcinoma [133], breast cancer [134], and endometrial carcinoma [135].

#### PTEN expression in cancer

PTEN mutations occur in both hereditary and somatic tumor syndromes and are responsible for a large percentage of human cancers. Hereditary PTEN mutations cause PTEN hamartoma tumors syndromes (PHTSs), which feature a variety of benign and malignant tumors [136]. Affected PHTS patients develop disorganized and hyperplastic cellular overgrowth, which eventually affects various tissues in the thyroid, breast, skin, and/or brain, and can also cause neurodevelopment disorders such as autism spectrum disorder [136]. In somatic cancers, including uterine corpus endometrial carcinoma (UCEC), breast cancer, prostate cancer, and glioblastoma, PTEN inactivation results in missense and nonsense mutation, mono- or bi-allelic deletion of the genomic locus or silencing through promotor methylation [137, 138].

Additionally, PTEN protein expression is either lost or reduced in 40% of primary breast carcinomas as assessed by the IHC [139]. Reduced PTEN expression can result in homozygous deletion of the PTEN gene locus or epigenetic silencing of the PTEN promotor [140, 141]. However, some studies observe a correlation between PTEN promoter hypermethylation and breast cancer while other studies do not conclude a correlation [141, 142].

Among 538 cases of clear cell renal cell carcinoma (ccRCC), 5% of patients carried the PTEN mutation. Patients with the PTEN mutation had poorer prognosis on survival, higher rates of metastasis, and disease recurrence compared to patients with WT-PTEN. Kurose et al. concluded that there is a prominent role of PTEN inactivation in ovarian carcinomas associated with increased pAkt [143] (Table 3).

**TABLE 3:** PTEN mutation/loss prevalence across major cancers.

Cancer type	Prevalence	Mutation type	Loss mechanism	Notes and references
Endometrial cancer	50–80%	Point mutation, deletion	LOH, promoter methylation missense mutations in the phosphatase domain, an in-frame deletion, and large insertion	Highest PTEN mutation rate of any solid tumor; early event in endometrioid subtype; present in 50–86% depending on microsatellite instability status [135, 144, 145]
Glioblastoma (GBM)	30–40%	Deletion, mutation	10q23 deletion, LOH	PTEN mutation rate 40–65% across GBM cohorts; mutations detected in 17–31% of primary GBMs [127, 146]
Prostate cancer (PCa)	17–61%	Deletion (homozygous)	10q23 CNA; hemizygous/homozygous deletion	Deletions present in 20.2% of hormone-naïve PCa rising to 40–50% in mCRPC; homozygous deletion in 12.1%; predominantly CNA (60–90%); prognostic for early PSA recurrence [146–148]
Breast cancer	8–47%	Point mutation, deletion	LOH, epigenetic silencing	Higher in TNBC and HER2+ subtypes; PTEN loss co-occurs with PIK3CA mutation; PTEN mutation or IHC loss present in majority of PI3K-altered cases [149]
Melanoma	10–25%	Deletion, mutation	10q23 loss, epigenetic mechanisms	PTEN loss co-occurs with BRAF V600E in ∼30%; PTEN deletion mediates intrinsic BRAF inhibitor resistance by maintaining PI3K/AKT signaling [150, 151]
Colorectal cancer (CRC)	5–41%	Point mutation	10q23 LOH, epigenetic mechanisms (promoter hypermethylation)	Frequently co-occurs with BRAF and KRAS mutations in CRC [152]
Lung cancer	0–59%	Deletion, mutation	Promoter methylation, protein loss, mutation	PTEN protein loss in lung cancer is not fully explained by genetic alterations, indicating that epigenetic and post-translational mechanisms also play key roles in regulating its expression [153]
Ovarian cancer	11–35%	Mutation, deletion	10q23 LOH	Concurrent mutations in ARID1A and PIK3CA drive ovarian clear-cell tumor development by enhancing pro-tumor inflammatory cytokine signaling [154–156]
Thyroid cancer	5–59%	Mutation, deletion	10q23.3 LOH, epigenetic and/or structural silencing mechanisms	PTEN mutation is a very rare mutation in thyroid nodules with no clear prognostic indicators [157–159]

Akt: protein kinase B, ARID1A: AT-rich interactive domain-containing protein 1A, BRAF: B-Raf proto-oncogene serine/threonine kinase, CNA: copy number alteration, CRC: colorectal cancer, GBM: glioblastoma multiforme, HER2+: human epidermal growth factor receptor 2 positive, IHC: immunohistochemistry, KRAS: kirsten rat sarcoma viral oncogene homolog, LOH: loss of heterozygosity, mCRPC: metastatic castration-resistant prostate cancer, PCa: prostate cancer, PI3K: phosphoinositide 3-kinase, PIK3CA: phosphatidylinositol-4, 5-bisphosphate 3-kinase catalytic subunit alpha, PSA: prostate-specific antigen, PTEN: phosphatase and tensin homolog, TNBC: triple-negative breast cancer, V600E: valine-to-glutamic acid substitution at codon 600, 10q23: chromosome 10q23 locus, 10q23.3: chromosome 10q23.3 locus.

#### PTEN-mediated signaling

The PTEN/AKT pathway is initiated when ligands (growth factors, cytokines, or hormones) bind RTKs which subsequently activates PI3Ks, lipid kinases that phosphorylate the 3′ hydroxyl of phosphatidylinositol. [160]. PI3Ks contain two domains: one P110 and one P85 domain. PI3K activation typically occurs through the binding of the P85 subunit or through the adapter molecules such as the insulin receptor substrate (IRS) proteins [160]. If RTKs are not present, PI3K can become activated by a GTP binding to the Ras protein [161]. Activated PI3K phosphorylates phosphatidylinositol-4,5-bisphosphate (PIP_2_) to produce PIP_3_. The role of PIP_3_ is to act as a dock for phospholipids where proteins can be recruited to the plasma membrane and subsequent activation of the signaling cascade [161]. From that point, PIP_3_ can either bind directly to the Akt or PDK1 protein. If PDK1 phosphorylates the binding site Thr308 of Akt, there will be partial activation, however, phosphorylation of Akt at Ser473 will stimulate full Akt activation [161].

Akt acts as a key signaling node because it is responsible for initiating and regulating the processes of multiple downstream cytoplasmic and nuclear targets [162]. Akt is interconnected with several signaling pathways including cyclin D1 [163], glycogen synthase kinase-3B (GSK3B) [164], forkhead [165], and Bcl-2–associated death promoter (BAD) [166]. Therefore, pAkt is responsible for modulating processes involving cell survival, progression, DNA repair, angiogenesis, and cellular migration [162]. Overexpression of this signaling pathway results in abnormal cell proliferation (oncogenesis) [167].

**FIGURE 5:**
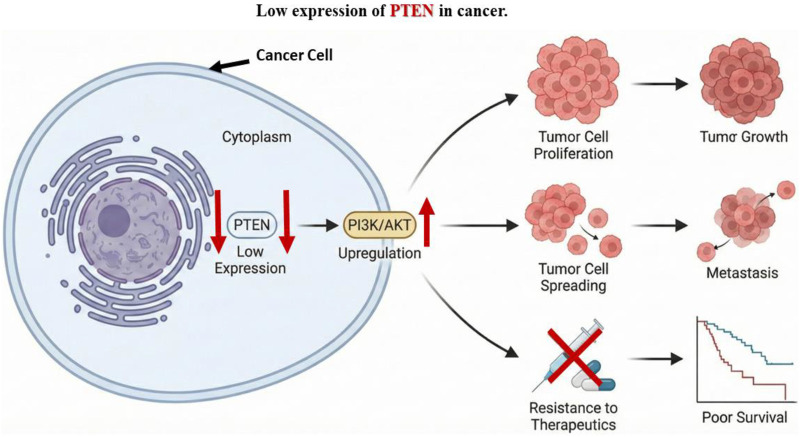
Low expression of PTEN in cancer. Schematic diagram illustrating the molecular and biological consequences of downregulation, mutation, or loss of phosphatase and tensin homolog (PTEN) in cancer cells. Under normal conditions, PTEN negatively regulates the PI3K/Akt signaling pathway by dephosphorylating phosphatidylinositol-3,4,5-trisphosphate (PIP_3_) to PIP_2_, thereby restraining Akt activation. Loss or functional impairment of PTEN removes this inhibitory control, leading to constitutive activation of the PI3K/Akt pathway, as indicated by directional arrows. Sustained PI3K/Akt signaling promotes tumor cell proliferation, survival, migration, and invasion, while inhibiting apoptotic responses and enhancing resistance to cytotoxic, targeted, and radiation-based therapies. Collectively, these signaling alterations drive tumor growth, metastatic progression, therapeutic resistance, and poor patient survival. This figure highlights the central role of PTEN as a tumor suppressor and gatekeeper of survival signaling in cancer. AKT: protein kinase B, PI3K: phosphatidylinositol 3-kinase, PTEN: phosphatase and tensin homolog.

PTEN dephosphorylates the lipid substrate PIP_3_ at the 3′ position converting PIP_3_ back to PIP_2_, thereby halting the phosphatidylinositol 3 (PI3)/Akt mitogenic signaling pathway. PTEN acts as the central negative regulator of PI3K by opposing its activity and dephosphorylating PIP_2_ into PIP_3_. A lack of PTEN leads to elevated levels of pAkt (Figure 5) [168].

#### Regulation of PTEN expression

##### Transcriptional regulation of PTEN expression

Positive regulators of PTEN include the early growth response protein 1 (EGR-1), tumor protein 53 (p53), active transcription factor 2 (ATF2), and peroxisome proliferator-activated receptor gamma (PPARγ) [169, 170].

EGR-1 binds to the PTEN 5′ UTR containing a functional GCGGGGGCG EGR-1 binding site. In addition, inducing EGR-1 by exposing cells to ultraviolet light upregulated the expression of PTEN mRNA and protein, thereby leading to apoptosis [171]. Similarly, p53 upregulates PTEN transcription by binding to the functional p53 binding element upstream of the PTEN promoter [172]. ATF2 bound to both site 1: (cctTGACGggtggg) and site 2: (ggcTGACGgccatt) in the PTEN promoter, however, the basal binding of ATF2 to site 2 was higher than site 1 [170]. Furthermore, activation of PPARγ by selective ligand upregulated PTEN expression in human macrophages [173]. On the other hand, NF-κB negatively regulates PTEN expression. p65 repressed the PTEN promoter and NF-κB activation was sufficient enough to inhibit PTEN expression [81].

Other transcription factors that were reported to inhibit PTEN expression in several cancer models were: mitogen-activated protein kinase kinase-4 (MKK4) and B-lymphoma Moloney murine leukemia virus (Mo-MLV) insertion region 1 (BMI1) [169]. In both mouse embryo fibroblast (MEF) cells and non-small cell lung cancer (NSCLC) cells, high MKK4 expression is correlated with low PTEN expression. Similarly, high PTEN expression was associated with a reduction in intracellular phosphoinositides, which are required for Akt activation [174]. MKK4 inhibited PTEN expression by nuclear translocation of p65 and activation of NF-κB [174]. Moreover, nuclear PTEN negatively regulates BMI1 expression through its C-terminal domain [175].

##### Epigenetic regulation of PTEN expression

Furthermore, lysine-specific demethylase 1 (LSD1), EZH2, and G9a were reported to epigenetically regulate PTEN expression. Knockdown of Myt1 by siRNA decreased the amount of LSD1 recruitment at the PTEN promoter, which indicated that nLSD1 directly binds to the PTEN promoter through Myt1. Neuro2a cell proliferation decreased as a result of LSD1 or Myt1 expression, which makes sense considering how PTEN negatively regulates cell proliferation [176]. Similarly, downregulated EZH2 and LSD1 could dramatically increase the expression level of PTEN. EZH2 and LSD1 can directly bind to the promoter regions of PTEN where it demethylates H3K27me3 [177].

A study by Bhat et al. identified PTEN to be a direct target of G9a. They were able to identify the PI3K signaling pathway as a target downstream of G9a. PTEN mRNA was significantly upregulated in G9a knockdown cells, indicating that PTEN mRNA is regulated by G9a in a methylation-dependent manner. G9a enrichment was evident at the PTEN promoter. The loss of PTEN expression by G9a resulted in reduced Akt activity and consequent proliferation [178].

##### Post-transcriptional regulation of PTEN expression

Multiple miRNAs—including miR-21, miR-22, miR-214, miR-205, miR-552, miR-106b, and miR-93—target the 3′ UTR of PTEN mRNA, with miR-21 inhibition increasing PTEN expression and its overexpression promoting tumor cell proliferation, migration, and invasion [179]. There is a highly significant inverse correlation between the abundance of PTEN and miR-22 [180]. miR-214 is upregulated following T cell activation, coinciding with a substantial reduction in PTEN mRNA in both CD4^+^ and CD8^+^ T cells [181]. Moreover miR-205 directly inhibited PTEN. miR-205 inhibited the luciferase reporter activity of WT PTEN 3′ UTR, but the inhibition was less changed for 3′ UTR with mutated binding sites [182]. Furthermore, PTEN mRNA was downregulated in miR-552 overexpression ovarian cancer (OV) cells, and there was a significant negative correlation between miR-552 and PTEN mRNA expressions in human OV tissues [183]. On a similar note, expression levels of miR-106b and miR-93 were detected by immunofluorescence (IF) staining. Upregulation of miR-106b and miR-93 in MCF-7 cells reduced PTEN expression. However, downregulation of miR-106b and miR-93 in MDA-MB-231 cells increased PTEN expression, all of which indicate how PTEN expression is inversely related to miR-106b and miR-93 [184].

On the other hand, there is increasing evidence of long noncoding RNAs (lncRNAs) regulating PTEN expression and the progression of human cancers. One type of lncRNA that was found to regulate PTEN expression in liver cancer stem cells was long noncoding RNA-downregulated in liver cancer (lnc-DILC) [185]. lnc-DILC is located at the chromosomal locus 13p24 and acts as a tumor suppressor for tumorigenesis and metastasis in liver and colorectal cancer. Lnc-DILC expression was remarkably decreased in ccRCC tissues in comparison to normal tissues, and a decrease in lnc-DILC expression was correlated with a larger tumor size [185]. PTEN mRNA levels were not affected by knockdown or overexpression of lnc-DILC; however, PTEN protein level was significantly increased after lnc-DILC overexpression. Therefore, it was concluded that lnc-DILC regulated PTEN expression at the post-translational level [185].

Xin et al. reported that lnc-HULC is upregulated in liver cancer tissues and negatively correlates with PTEN protein expression, indicating a strong translational level inverse relationship [186]. lnc-GAN1 is downregulated in lung tumors, with low expression correlating with poorer overall and disease-free survival (DFS), and that lnc-GAN1 upregulates PTEN mRNA and protein levels [187]. Lnc-FTX enhances PTEN expression induced by Angiotensin II (Ang II), suppressing PI3K/AKT signaling and reducing Ang II–induced cardiac myocyte hypertrophy in mice [188]. lnc-MIR17HG mRNA expression level was decreased in acute myeloid leukemia (AML), supporting a potential tumor-suppressive role, and regulation of PTEN [189].

##### Post translational regulation of PTEN expression

Furthermore, there are various enzymes responsible for phosphorylation of PTEN on the C-terminal domain including casein kinase 2 (CK2), GSK3, and Rho-associated (RhoA) protein kinase (ROCK).

CK2 phosphorylates PTEN at C-terminal residues Ser370, Ser380, Ser385, Thr382, and Thr383, with Ser370 and Ser385 being critical for CK2-mediated phosphorylation and overall PTEN protein stability [190]. Similarly, CK2 phosphorylates Ser370 and Ser385 *in vitro* while Thr366 was also phosphorylated, but to a lower extent [191].

GSK3 phosphorylates serine or threonine residues, though it has been reported that there is a modestly higher activity against serine [192]. In cells with reduced levels of both GSK3 isoforms, phosphorylation of PTEN at Thr366 was much reduced. This concluded that GSK3 does phosphorylate Thr366 in intact cells [193]. ROCK, a downstream effector of RhoA, upregulates PTEN activity, and its inhibition reduces PTEN activity, thereby increasing Akt phosphorylation [194].

#### Role of PTEN, RKIP, and GSK-3β in resistance

##### Resistance to chemotherapy

GSK-3 inhibition by small molecules, particularly 9-ING-41, suppresses breast cancer cell growth, spares non-tumorigenic cells, and potentiates irinotecan (CPT-11)-mediated tumor reduction *in vivo* [50]. Altogether, their results suggest that GSK3 is a promising therapeutic approach to overcome chemoresistance in human breast cancer [50]. GSK3β modulates PTEN and Akt phosphorylation, regulating cell migration, apoptosis, and chemoresistance in breast cancer via the PTEN/PI3K/Akt axis [195].

The modulation of PTEN expression or mutation in many cancers resulted in the activation of the PI3K/Akt pathway that regulates tumor cell proliferation, invasion, and resistance to cytotoxic drugs [196]. Inactivation of PTEN contributed to tumor cells unresponsiveness to cytotoxic chemotherapeutic drugs. The expression level of miRNA-17-5p was elevated in patients who were resistant to chemotherapy. [197]. miRNA-193-3p was upregulated in both gastric cancer cell lines and human gastric tumors resistant to 5-fluorouracil (5-FU) both *in vitro* and *in vivo* miRNA-141-3p was overexpressed in resistant esophageal cancer cells from patients. [198].

Overexpression of cancer susceptibility candidate 2 (CASC2) sensitized the tumor cells to TMZ [199]. In addition, CASC2 upregulated PTEN protein and inhibited pAkt protein. The upregulation of PTEN by CASC2 was via the direct inhibition of miRNA-181 [197]. long noncoding RNA-activated in renal cell carcinoma with sunitinib resistance (lncARSR) was upregulated in HCC and associated with large tumor size and poor prognosis. The overexpression of lncARSR augmented the resistance of HCC cells to doxorubicin (DOX) *in vitro* and *in vivo* [200]. miR-96-5p targets PTEN expression and affected the chemosensitivity and radiosensitivity of head and neck squamous cell carcinoma (HNSCC) cells [201]. Also, miR-96-5p activated the PI3K/Akt/mTOR pathway. miR-96-5p could serve as a biomarker for chemo-radio sensitivity [198]. miR-21 interaction with PTEN regulated the sensitivity of LUAD cells to 5-FU-mediated cytotoxicity. Overexpression of miR-21 inhibited 5-FU-mediated apoptosis in cancer cells [202]. Overexpression of PTEN resulted in the induction of apoptosis (intrinsic mitochondrial pathway), inhibition of cell proliferation in breast cancer cells, and reversing the chemoresistance [203]. PTEN was a direct target miR-4461 in OV. There was an inverse correlation between the expression of PTEN and miR-4461 in OV tissues. In addition, miR-4461 was in part responsible for the resistance of OV cells to cisplatin [204]. PTEN–deficient tumors rely on ATM kinase for DNA damage repair, and low-dose ATM inhibitor synergizes with radiation to overcome resistance in lung cancer models [205].

RKIP functions as an apoptosis inducer, thereby causing the re-sensitization of resistant tumors to chemotherapy [206]. The transcription factor NF-κB promotes tumor resistance to chemotherapy and immunotherapy by inducing the expression of anti-apoptotic gene products related to Bcl-2 and regulating/decreasing the expression of death receptors (DRs) [207]. Furthermore, certain small molecules including proteasome inhibitor, NPI-0052, bortezomib, and nitric oxide (NO) donor DETA/NO are able to sensitize multiple cancer cell lines to chemotherapy-related apoptosis through NF-κB and Snail inhibition and RKIP induction [207, 208]. Proteasome inhibitor NPI-0052 and bortezomib inhibited NF-κB and its downstream target, Snail (a repressor of RKIP), resulting in the depression of RKIP [206]. The treatment of human prostate cancer cell lines by NPI-0052 or NO donor DETA/NO resulted in the reversal of tumor cell EMT, migration, and invasion [207]. It was suggested that this occurred through RKIP-mediated NF-κB inhibition as well as the subsequent suppression of the EMT inducer, Snail [207]. On the other hand, NF-κB constitutive activity has also been associated with the cause of adaptive tumor resistance to ionizing radiation [207, 209]. Silencing Snail or RKIP ectopic induction has direct effects that suppress the expression of anti-apoptotic proteins from the Bcl-2 family. This supports the conclusion that RKIP and the NF-κB/Snail module have opposing roles in the regulation of tumor resistance.

##### Resistance to immunotherapy

Immune checkpoint blockade (ICB) is an important and increasingly popular approach in immunotherapy while many cancers still remain insensitive to ICB [25]. GSK3 inhibitors, including GSK3i and SB415286, were observed to downregulate transcription of programmed cell death protein 1 (PD-1) in CD8^+^ T cells [210]. This connects to PTEN because GSK3β can mediate the phosphorylation of Akt and PTEN to promote chemoresistance [211]. Identifying GSK3 inhibitors, such as GSK3i and SB415286, is important because they do not only inhibit the PTEN/PI3K/Akt signaling pathway, but they also downregulate transcription of PD-1 in CD8^+^ T cells, which activates CD8 cytotoxic T cells and decreases tumor proliferation, invasion, and cell cycle progression [212].

By decreasing PTEN expression, the percentage of lysed tumor cells was significantly reduced. The loss of PTEN can cause resistance to T cell-mediated immune responses and resistance to immunotherapy in melanoma [213]. The PI3Kβ inhibitor enhanced the efficacy of immunotherapy in melanomas with PTEN loss [213].

Additionally, a PTEN mutations in the phosphatase domain are correlated with PTEN loss of function, resulting in resistance to PD-1 blockade [214].Furthermore, mRNA delivery by polymeric nanoparticles (NPs) can effectively induce the expression of PTEN when it is mutated in melanoma cells and lost in prostate cancer cells. Their results showed that these PTEN mRNA cells not only restored the susceptibility of tumor cells to death but also led to the release of damage-associated molecular patterns (DAMPs) [215]. The combination of PTEN mRNA NP with an immune checkpoint inhibitor (ICI) antibody [anti-programmed cell death ligand 1 (PDL1)] results in a highly potent anti-tumor effect when observed in a subcutaneous mutated PTEN model from melanoma and PTEN-null prostate cancer model [215].

Dendritic cells (DCs) from elderly (65–90 years) exhibit higher PTEN levels and increased NF-κB activation, correlating with enhanced reactivity to human DNA compared with young (20–35 years) subjects [216]. In the dysregulated NF-κB/Snail/YY1/RKIP loop, the overexpression of NF-κB, Snail, and YY1 has led to the maintained downregulation of RKIP. The maintained hyperactivation of NF-κB and its targets, Snail and YY1, results in cell resistance and insensitivity to both chemo- and immune-therapeutic drugs [217]. In addition to RKIP inhibiting anti-proliferative and tumor suppressor functions, it also acts as an anti-resistant factor [206].

The role of RKIP in the reversal of immune resistance was examined through the RKIP disruption loop by Bonavida [206]. Both NK cells and cytotoxic T lymphocytes (CTLs) mediate their killing mechanisms by both necrotic and apoptotic mechanisms. The necrotic mechanism mediates its cell death mechanism through the perforin/granzyme system by perforating holes on the cell membrane. This perforation results in changes to the osmotic pressure, lysis of the cells, as well as apoptosis [218]. On the other hand, the apoptotic mechanism begins with the interaction of ligands on the lymphocytes such as TNF-α, Fas ligand (FasL), and TRAIL with the corresponding receptors (TNFR-1/2, Fas, DR4, and DR5). The contact between sensitive target cells and cytotoxic lymphocytes leads to the activation of the apoptotic pathway, thereby resulting in cell death [206].

The sensitization of Fas-resistant tumor cells to FasL cytotoxicity was achieved via the treatment of tumor cells with a NO donor that resulted in the upregulation of Fas on the tumor cells and sensitization to apoptosis. The underlying mechanism was investigated, and it was found that NO inhibited the Fas transcription repressor, YY1 [219]. In addition, upregulation of RKIP in tumor cells also resulted in the upregulation of Fas via RKIP-mediated inhibition of NF-κB and downstream YY1 [220]. Further studies also revealed that YY1 represses the TRAIL receptor DR5 and its inhibition by NO or by the upregulation of RKIP resulted in the sensitization of TRAIL-resistant tumor cells to TRAIL apoptosis [221, 222].

Immune checkpoint inhibitors (ICIs), including antibodies targeting PD-1, PD-L1, and CTLA-4, have transformed cancer therapy; however, primary and acquired resistance remain major challenges [223]. Emerging evidence highlights the role of tumor-intrinsic signaling pathways, particularly those involving GSK-3β and PTEN, in regulating antitumor immunity and response to immunotherapy.

GSK-3β modulates immune checkpoint regulation at both transcriptional and post-transcriptional levels. Its activity promotes PD-1 expression, whereas pharmacological inhibition reduces PD-1 transcription and enhances CD8^+^ T-cell and NK cell function, providing a rationale for combining GSK-3β inhibitors with PD-1/PD-L1 blockade [224].

PTEN is similarly critical, as its loss is associated with immune-excluded tumor microenvironments, reduced T-cell infiltration, and resistance to ICIs [225]. Mechanistically, PTEN deficiency sustains PI3K/Akt signaling and promotes immunosuppressive programs that facilitate immune evasion. Clinically, low PTEN expression correlates with poor response to immunotherapy [226]. Importantly, PTEN and GSK-3β pathways converge to regulate immune escape and therapeutic resistance. PTEN loss enhances Akt signaling, thereby altering GSK-3β activity, while GSK-3β regulates transcription factors involved in immune checkpoint control [227]. Together with RKIP downregulation and NF-κB activation, these alterations establish a tumor-intrinsic resistance state that limits the efficacy of immune checkpoint blockade.

The GSK-3β/RKIP/PTEN axis coordinates signaling (PI3K/AKT, NF-κB, β-catenin) that sculpts stromal and immune compartments. Its dysregulation shifts cytokine profiles, favors immunosuppressive cell states, promotes fibroblast/stromal remodeling, and reduces immunotherapy sensitivity. GSK-3 plays an important role in immune and hematological cell regulation through its constitutive kinase activity, where it modulates multiple signaling pathways by phosphorylating key regulatory proteins, including oncogenic and immune-related mediators such as β-catenin and c-Myc, thereby influencing cellular proliferation, survival, and immune-associated functions [56]. RKIP functions as an important immunomodulator by regulating inflammatory and immune signaling pathways, particularly through inhibition of NF-κB and ERK/MAPK signaling, modulation of pro-inflammatory cytokine production, and control of immune-cell infiltration within the tumor microenvironment, thereby influencing tumor progression, immunosurveillance, and sensitivity to immunotherapeutic agents [195].

Loss of PTEN in stromal fibroblasts was shown to accelerate mammary tumor initiation, progression, and malignant transformation by promoting extracellular matrix remodeling, immune cell infiltration, and angiogenesis through activation of the PTEN-Ets2 (v-ets erythroblastosis virus E26 oncogene homolog 2) signaling axis [228]. PTEN exerts critical tumor cell–non-autonomous functions within the tumor microenvironment, where stromal PTEN inactivation promotes cancer progression through dysregulation of intracellular communication pathways and altered signaling interactions between stromal and tumor cells, ultimately contributing to more aggressive disease behavior and poorer clinical outcomes [78].Mechanistically, PTEN loss in fibroblasts downregulates miR-320, which directly targets Ets2; the resulting Ets2 upregulation orchestrates a pro-oncogenic secretome containing matrix metallopeptidase 9 (MMP9) and elastin microfibril interfacer 2 (EMILIN2) that enhances tumor cell invasion and angiogenesis [229].

## Cross-talk signaling pathways between RKIP, GSK-3β and PTEN

### Indirect regulation between MAPK and PI3/Akt pathway

There is an indirect regulation between RKIP and PTEN because B-RAF mutations are involved in the dysregulation of the MAPK and PI3K/Akt pathways. B-RAF mutations are present in up to 70% of melanoma cases [230]. Therefore, it is important to determine if these signaling crosstalk pathways are related indirectly and/or directly as they can offer novel targets for therapeutic interventions. In fact, the most promising treatments for melanoma seem to target mutations involved in the RAF-MEK and PI3K pathways. B-RAF is a member of the RAF family proteins that are involved in the signal transduction cascade involved in the regulation of apoptosis, proliferation, and transformation to cancerous states [231].

**FIGURE 6:**
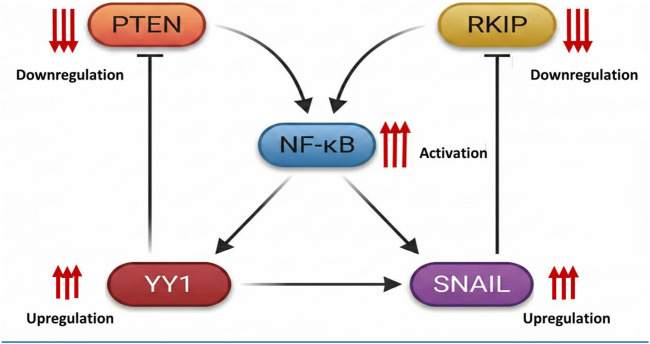
Schematic diagram depicting cross-talk signaling pathways between RKIP and PTEN via the NF-κB/Snail/YY1 loop. Schematic diagram illustrating the dysregulated NF-κB/Snail/YY1 signaling loop that functionally links RKIP and PTEN in cancer cells. Activation of the IκB kinase (IKK) complex leads to nuclear translocation and activation of NF-κB, which subsequently induces the expression of downstream transcription factors Snail and Yin Yang 1 (YY1), as indicated by activating arrows. Snail acts as a direct transcriptional repressor of RKIP, resulting in diminished RKIP expression and loss of its inhibitory control over upstream signaling pathways. Suppression of RKIP, in turn, sustains and amplifies NF-κB hyperactivation, establishing a positive feed-forward loop. Concomitantly, NF-κB–driven upregulation of YY1 leads to transcriptional repression of PTEN, thereby promoting PI3K/Akt pathway activation and enhancing tumor cell survival. Collectively, this interconnected regulatory circuit drives epithelial–mesenchymal transition, resistance to chemotherapy and immunotherapy, and aggressive tumor progression. Directional arrows indicate pathway activation, whereas blunt-ended lines denote transcriptional repression, highlighting how coordinated loss of RKIP and PTEN cooperatively reinforces oncogenic and therapy-resistant signaling states. PTEN: phosphatase and tensin homolog, RKIP: Raf kinase inhibitor protein, SNAIL: zinc finger transcription factor Snail family transcriptional repressor 1, YY1: Yin Yang 1 transcription factor.

As shown in this review, RKIP negatively regulates the MAPK pathway via the inhibition of B-RAF activity. The inhibition of B-RAF by RKIP results in a downregulation of all metastatic pathways associated with B-RAF (Figure 6).

### Indirect regulation via NF-κB/Snail/YY1 loop

There was another indirect relationship between RKIP and PTEN via the dysregulated NF-κB/Snail/YY1 loop expressed in cancer. RKIP’s role in inhibiting NF-κB is paramount in controlling the proliferation of cancer cells [79]. Hyperactivation of the NF-κB pathway promotes overexpression of YY1 and Snail, leading to RKIP suppression and maintenance of a positive feedback loop that enhances metastatic signaling, whereas RKIP overexpression disrupts this circuit by inhibiting NF-κB and its downstream effectors YY1 and Snail [116].

**FIGURE 7:**
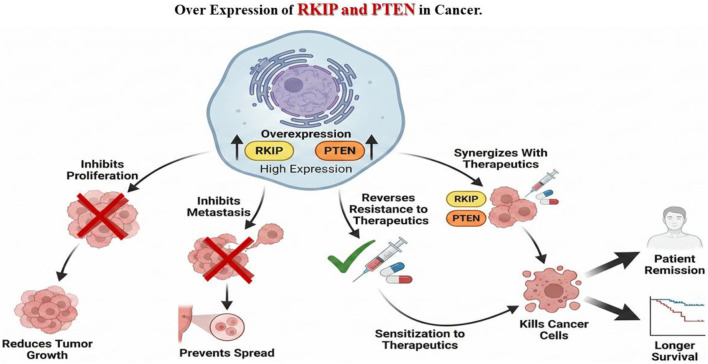
Role of RKIP and PTEN in cancer. Schematic diagram illustrating the tumor-suppressive and chemosensitizing effects of RKIP and PTEN overexpression in cancer cells. When RKIP expression is preserved or restored, it inhibits the Raf-1/MEK/ERK (MAPK) pathway, thereby suppressing oncogenic signaling associated with cell proliferation, migration, and metastasis. In parallel, PTEN acts as a key negative regulator of the PI3K/Akt signaling pathway by limiting Akt activation, resulting in reduced cell survival signaling and enhanced apoptotic responsiveness. The coordinated activity of RKIP and PTEN leads to inhibition of tumor cell proliferation, suppression of metastatic spread, and increased sensitivity to cytotoxic therapeutic agents. Enhanced therapeutic sensitivity allows effective induction of tumor cell death, which translates into tumor regression, disease control, and improved clinical outcomes. This figure highlights how restoration or maintenance of RKIP and PTEN expression shifts signaling balance toward growth suppression, therapy responsiveness, and prolonged patient survival. PTEN: phosphatase and tensin homolog, RKIP: Raf kinase inhibitor protein.

There is another indirect relationship between RKIP and PTEN because PTEN expression is low in most cancers, in part by NF-κB and YY1. PTEN is either downregulated or absent in most cancer cells because NF-κB hyperactivation and overexpression of YY1 (PTEN inhibitor) decrease PTEN expression [232]. When PTEN is inhibited, the Akt/PI3K pathway becomes activated and results in tumor cell survival, growth, and resistance [79]. Essentially, the hyperactivation of NF-κB and YY1 results in the downstream inhibition of RKIP in the MAPK pathway and PTEN in the PI3K/Akt. Therefore, we believe in the dysregulated loop, there exists a direct relationship between both gene products or pathways (Figure 7).

### The PI3K/PTEN/Akt/mTOR pathway and GSK-3

**FIGURE 8:**
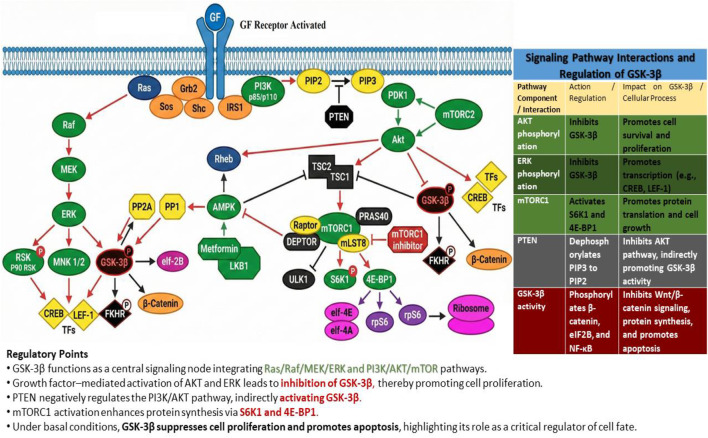
Interactions of GSK-3 with the Ras/PI3K/PTEN/Akt/mTOR and Ras/Raf/MEK/ERK Pathways. Some of the regulatory interactions between GSK-3 and Ras/PI3K/PTEN/Akt/mTOR and Ras/Raf/MEK/ERK pathways are indicated. An activated growth factor receptor is indicated in blue. Ras and Rheb are indicated in dark blue ovals. IRS1, Shc, Grb2, Sos and beta-catenin are indicated in orange ovals. Kinases are indicated in green ovals with the exception of GSK-3β which is indicated in a red oval. The p85 regulatory subunit of PI3K is indicated in a green oval. The phosphatases which inhibit steps in this pathway are indicated in black octagons. The phosphatases PP2A and PP1 which may activate GSK-3 are indicated in yellow octagons. NF1, TSC1 and TSC2 are indicated in black squares. PIP2 and PIP3 are indicated in yellow ovals. The mTORC1 inhibitor is indicated in a red octagon. The AMPK activator Metformin is indicated in a green octagon. mTOR interacting proteins which positively regulate mTOR activity are indicated in yellow ovals. mTOR interacting proteins which negatively regulate mTOR activity are indicated in black ovals. Transcription factors activated by either ERK or Akt phosphorylation are indicated in yellow diamonds. The FKHR transcription factor that is inactivated by Akt phosphorylation is indicated by a black diamond and a white P in a black circle. FKHR is also activated by GSK-3βphosphorylation which is indicated by a white P in a red circle. beta-catenin is indicated in an orange oval. mRNA initiation factors and proteins associated with the ribosome are indicated in magenta ovals. mTORC1 phosphorylates the unc-51-like kinase 1 (ULK1) which results in the suppression of autophagy. ULK1 is indicated in a black oval. In contrast, AMPK activates both ULK1 and autophagy as well as TSC activity. Proteins involved in the regulation of translation are indicated in purple ovals. Red arrows indicate activating events in pathways. Black arrows indicate inactivating events in pathway. Activating phosphorylation events are depicted in red circles with Ps with a black outlined circle. Inactivating phosphorylation events are depicted in black circles with Ps with a red outlined circle. This figure is provided to give the reader an idea of the complex interactions of GSK-3 with various signaling molecules in the Ras/PI3K/PTEN/Akt/mTOR and Ras/Raf/MEK/ERK pathways which are key in regulating cellular proliferation survival and often become dysregulated in cancer. AMPK: AMP-activated protein kinase, Akt: protein kinase B, β-Catenin: Beta catenin, CREB: cAMP response element-binding protein, DEPTOR: DEP domain-containing mTOR-interacting protein, elf-2B: eukaryotic translation initiation factor 2B, elf-4A: eukaryotic translation initiation factor 4A, elf-4E: eukaryotic translation initiation factor 4E, ERK: extracellular signal-regulated kinase, FKBP38: FK506-binding protein 38, FKHR: Forkhead in rhabdomyosarcoma, GF: growth factor, Grb2: growth factor receptor-bound protein 2, GSK 3β: glycogen synthase kinase 3 Beta, IRS1: insulin receptor substrate 1, LEF-1: lymphoid enhancer-binding factor 1, LKB1: liver kinase B1, MNK 1/2: MAP kinase-interacting kinases 1 and 2, mLSTB: mammalian lethal with SEC13 protein 8, mTOR: mechanistic target of rapamycin, mTORC1: mTOR complex 1, mTORC2: mTOR complex 2, NF1: neurofibromin 1, P90 RSK: 90-kDa ribosomal S6 kinase, PDK1: 3-phosphoinositide-dependent protein kinase-1, PIP2: phosphatidylinositol 4,5-bisphosphate, PIP3: phosphatidylinositol 3,4,5-trisphosphate, p85PI3K: regulatory subunit p85 of phosphoinositide 3-kinase, p110PI3K: catalytic subunit p110 of phosphoinositide 3-kinase, PP1: protein phosphatase 1, PP2A: protein phosphatase 2A, PRAS40: proline-rich Akt substrate of 40 kDa, PTEN: phosphatase and tensin homolog, Ras: rat sarcoma viral oncogene homolog, Rictor: rapamycin-insensitive companion of mTOR, RKIP: Raf kinase inhibitor protein, rpS6: ribosomal protein S6, Shc: Src homology 2 domain-containing transforming protein, SIN1: SAPK interacting protein 1, SOS: son of sevenless, TSC1: tuberous sclerosis complex 1, TSC2: tuberous sclerosis complex 2, ULK1: Unc-51 like autophagy activating kinase 1.

The PI3K/PTEN/Akt/mTOR pathway and GSK-3 can cross-regulate each other at various points. Some of the interactions between GSK-3 and the PI3K/PTEN/Akt/mTOR pathway are presented in Figure 8. The PI3K/PTEN/mTOR pathway is involved in cancer initiation, metastasis, drug resistance, and sensitivity to therapy [233, 234]. PI3K p110 catalytic subunit (PIK3CA) mutations may be driver mutations in certain cancers responsible for metastasis [235]. Novel PI3K-alpha inhibitors have been isolated and they inhibit metastasis [235]. Most PI3K inhibitors are cytostatic rather than cytotoxic and it has been questioned whether treatment with a single PI3K inhibitor will be effective [236]. The PI3K/PTEN/Akt/mTORC1 pathway is important in c-Myc expression in Burkitt’s lymphomagenesis in germinal center B cells [237]. The PI3K/PTEN/Akt/mTORC1 pathway is also an emerging target for mantle cell lymphoma as this cascade is upregulated in this cancer [238]. Disruption of PTEN and TP53 activity in the thyroid has recently been shown to result in anaplastic thyroid carcinomas in murine models [239]. Recently it has been shown that reduction of PIK3CA or PI3K p85 regulatory subunit (PIK3RA) cell proliferation impedes proliferation, migration, and invasion in glioblastoma multiforme cells [240]. In contrast to PIK3CA, PIK3CB (PI3K p110-β catalytic subunit) is oncogenic in its WT configuration when it is overexpressed in certain conditions. PIK3CB can act like an oncogenic mutant of PIK3CA [241].

### Liver kinase B1 (LKB1)/AMPK network interactions with GSK-3 and PI3K/Akt/mTORC1 pathway

The LKB1/AMP activated protein kinase (LMPK) pathway is now recognized to play important roles in cancer and other diseases [242]. Activation of the LKB1/AMPK signaling pathway by metformin or 5-aminoimidazole-4-carboxamide 1-β-D-ribonucleotide (AICAR) induces tumor cell cycle arrest, apoptosis, or autophagy and suppresses mTORC1-mediated translation, including cancer-initiating cells [23, 243]. Metformin also suppresses proliferation of T-cell acute lymphoblastic leukemia (T-ALL) via inhibition of mTORC1, inducing autophagy and apoptosis, blocking mRNA translation, and targeting leukemia-initiating cells while exhibiting lower toxicity toward normal CD4^+^ T-lymphocytes [244]. AMPK is also important in BCR-ABL-induced chronic myeloid leukemia (CML) and AMPK pathway activators may prove useful as combination drug therapy (with BCR-ABL inhibitors) for this disease [242]. AMPK can also be activated by rapamycin [245].

LKB1 is an important tumor suppressor and gatekeeper mutations of LKB1 cause the rare Peutz-Jeghers syndrome (PJS) which is a cancer-prone syndrome [246]. LKB1 is a critical regulator of cell polarity and energy/metabolism control and exerts it vast effects via diverse effectors [247]. Inhibiting mTORC1 activity by drugs such as metformin and other drugs (including rapamycin) may not only aid in the treatment of diabetics, but also improve cancer therapies and increase longevity [248]. Berberine inhibits growth of drug-resistant breast cancer cells, potentially via the LKB1/AMPK signaling pathway, with heightened sensitivity in neutrophil gelatinase-associated lipocalin (NGAL)-overexpressing cells [249].

### Integration of p53 signaling with the GSK-3β/PTEN axis

The tumor suppressor p53 functionally intersects with PTEN and GSK-3β to coordinate cell fate, genomic stability, and therapeutic response. p53 transcriptionally upregulates PTEN, forming a feed-forward loop that suppresses PI3K/Akt signaling and promotes tumor-suppressive outcomes [250]. Through inhibition of Akt, PTEN indirectly maintains GSK-3β activity, enabling regulation of apoptosis and cell-cycle progression [227].

GSK-3β, in turn, modulates p53 stability and activity through direct phosphorylation, influencing its transcriptional function and interaction with regulators such as MDM2. Disruption of this p53–PTEN–GSK-3β axis, commonly through PTEN loss or p53 mutation, leads to sustained Akt activation, impaired apoptosis, and therapeutic resistance [251].

Overall, this interconnected network highlights the importance of coordinated tumor suppressor signaling in determining cancer progression and treatment response.

Although GSK-3β, RKIP, and PTEN regulate distinct signaling nodes, growing evidence supports their integration into a coordinated tumor-suppressive network that governs key oncogenic pathways, including PI3K/Akt, MAPK/ERK, Wnt/β-catenin, and NF-κB signaling [252] (Table 4).

**TABLE 4:** Biomarker co-associations: PTEN/RKIP/GSK-3β and co-occurring alterations.

Cancer type	PTEN loss + co-alteration	RKIP loss + co-alteration	pGSK-3β + co-alteration	Clinical/Prognostic significance and references
Prostate cancer	PTEN loss + ERG fusion (∼40%); PTEN loss + AR amplification [148]	RKIP↓ + Snail↑ [84]	pGSK-3β + nuclear AR↑ [253]	PTEN loss + ERG: Predicts aggressive disease and poor prognosis; RKIP loss provides metastatic phenotype; all three alterations converge in CRPC on PI3K/AR/NF-κB axes. Akt inhibition (ipatasertib + abiraterone) specifically benefits PTEN-loss subgroup (HR 0.77) [84, 148, 254]
Breast cancer	PTEN loss + PIK3CA mutation (partial overlap) in TNBC [149]	RKIP↓ + EZH2↑ + CXCR4↑ [82]	pGSK-3β + c-Myc↑ [255]	EZH2 simultaneously drives RKIP silencing (PRC2/H3K27me3) and multiple other tumor suppressor repression events; PTEN + PIK3CA co-alteration does not additively increase PI3K activation [99, 149]
Colorectal cancer	PTEN loss + APC mutation + KRAS mutation (triple hit) [256]	RKIP↓ + β-catenin nuclear↑ [257]	pGSK-3β + APC loss → WNT↑ [41]	Both RKIP loss and pGSK-3β elevation converge on β-catenin stabilization; triple-hit tumors show aggressive phenotype; meta-analysis confirms RKIP loss as independent poor-prognosis marker in CRC (HR 0.55 for OS) [101, 102]
Glioblastoma	PTEN del + EGFR amplification + CDK4 amplification [258]	RKIP↓ + PTEN co-deletion [7]	pGSK-3β + EGFR↑ + TMZ resistance [259]	EGFR amplification + PTEN loss = canonical GBM profile; simultaneous RKIP loss and pGSK-3β elevation maximize PI3K/RAS signal convergence; tideglusib sensitizes GBM to TMZ in preclinical models [33, 127, 260]
Melanoma	PTEN loss + BRAF V600E (∼30%) [150]	RKIP↓ + SNAI2↑ + BRAF↑(303)	pGSK-3β + MITF dysregulation [261]	PTEN loss predicts intrinsic vemurafenib resistance; RKIP loss further sustains ERK signaling after BRAF inhibition; combination PI3K inhibitor + BRAF inhibitor restores sensitivity in PDX preclinical models [105, 150, 151, 262]
Multiple myeloma	PTEN mutations are uncommon [263]	↑ inactive RKIP + Bcl-2 + DR5 (101)	pGSK-3β + bortezomib resistance [264]	RKIP–NF-κB axis in MM is independent of RAF/MEK; phosphorylated (inactive) RKIP maintains bortezomib resistance; reactivation with PKC inhibitor bisindolylmalemide restores drug sensitivity in MM cell lines [106, 107]

APC: adenomatous polyposis coli, AR: androgen receptor, Bcl-2: B-cell lymphoma 2, BRAF: B-Raf proto-oncogene serine/threonine kinase, CDK4: cyclin-dependent kinase 4, CRC: colorectal cancer, CRPC: castration-resistant prostate cancer, CXCR4: C-X-C chemokine receptor type 4, del: deletion, DR5: death receptor 5, EGFR: epidermal growth factor receptor, ERG: ETS-related gene, ERK: extracellular signal-regulated kinase, EZH2: enhancer of zeste homolog 2, GBM: glioblastoma multiforme, H3K27me3: trimethylation of histone H3 lysine 27, HR: hazard ratio, MITF: microphthalmia-associated transcription factor, MM: multiple myeloma, NF-κB: nuclear factor kappa-light-chain-enhancer of activated B cells, OS: overall survival, PDX: patient-derived xenograft, PI3K: phosphoinositide 3-kinase, PIK3CA: phosphatidylinositol-4, 5-bisphosphate 3-kinase catalytic subunit alpha, PKC: protein kinase C, PRC2: polycomb repressive complex 2, PTEN: phosphatase and tensin homolog, RAF: rapidly accelerated fibrosarcoma kinase, RAS: rat sarcoma viral oncogene homolog, RKIP: raf kinase inhibitory protein, SNAI2: snail family transcriptional repressor 2, TMZ: temozolomide, TNBC: triple-negative breast cancer, V600E: valine-to-glutamic acid substitution at codon 600, WNT: wingless/integrated signaling pathway.

PTEN serves as a primary upstream gatekeeper of the PI3K/Akt pathway by dephosphorylating PIP3 and limiting Akt activation [265]. Through this function, PTEN indirectly regulates GSK-3β activity, as Akt-mediated phosphorylation of GSK-3β at Ser9 alters its substrate specificity and signaling role. Loss of PTEN therefore results in sustained Akt activation, dysregulated GSK-3β signaling, and enhanced survival, metabolic reprogramming, and resistance pathways [266]. In parallel, RKIP negatively regulates MAPK/ERK signaling by inhibiting Raf-1, thereby constraining proliferative and anti-apoptotic signals that would otherwise synergize with PI3K/Akt activation [87].

At the transcriptional level, GSK-3β acts as an integrator of these upstream inputs, modulating key effectors such as NF-κB, β-catenin, Snail, cyclin D1, and c-Myc [267]. RKIP further intersects with GSK-3β-dependent transcriptional control through suppression of NF-κB and Snail-driven EMT programs, while PTEN loss potentiates these transcriptional responses by reinforcing Akt-dependent signaling dominance [268]. Importantly, feedback and feed-forward interactions among these pathways—particularly involving NF-κB, Snail, and YY1—create a dynamic regulatory loop in which disruption of PTEN or RKIP amplifies oncogenic GSK-3β outputs rather than simply altering its activity status.

**FIGURE 9:**
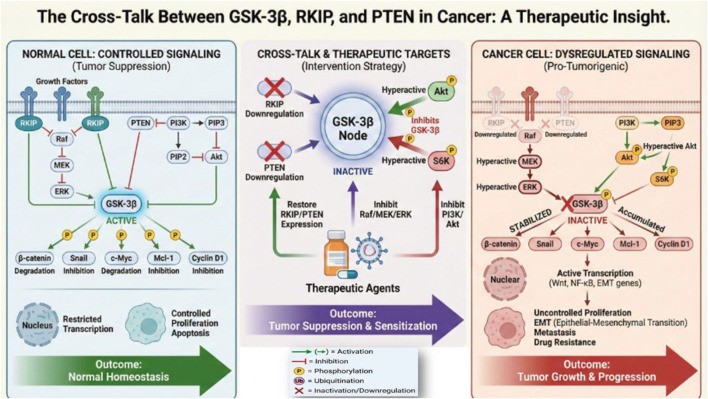
Schematic Overview of the Regulatory Crosstalk Between GSK-3β, RKIP, and PTEN in Normal and Malignant Cells with Therapeutic Implications. This figure illustrates the differential regulation of signaling networks centered on GSK-3β, RKIP, and PTEN in normal versus cancerous cells. In normal cells (left panel), RKIP suppresses the Raf/MEK/ERK cascade, while PTEN inhibits the PI3K/AKT pathway, maintaining GSK-3β in an active state and promoting tumor suppression. In cancer cells (right panel), downregulation of RKIP leads to disinhibition of MEK/ERK signaling and aberrant crosstalk with the PTEN/AKT axis, resulting in GSK-3β inactivation. This shift drives uncontrolled proliferation, epithelial–mesenchymal transition (EMT), metastasis, and drug resistance. The diagram highlights key nodes for therapeutic intervention, including restoration of RKIP function or targeting downstream effectors (e.g., AKT, ERK) to re-establish controlled signaling. Akt: protein kinase B, c-Myc: Myc proto-oncogene protein, EMT: epithelial-mesenchymal transition, ERK: extracellular signal-regulated kinase, GSK-3β: glycogen synthase kinase 3 beta, MEK: mitogen-activated protein kinase, Mcl-1: induced myeloid leukemia cell differentiation protein Mcl-1, NF-kB: nuclear factor kappa-light-chain-enhancer of activated B cells, PI3K: phosphoinositide 3-kinase, PTEN: phosphatase and tensin homolog, PTPN1: protein tyrosine phosphatase non-receptor type 1, PTPN2: protein tyrosine phosphatase non-receptor type 2, PTPN3: protein tyrosine phosphatase non-receptor type 3, Raf: rapidly Accelerated fibrosarcoma kinase, RKIP: raf kinase inhibitor protein, RTK: receptor tyrosine kinase, S6K: ribosomal protein S6 kinase, Wnt: wingless-related integration site.

At a systems level, this triad functions as a signaling rheostat that determines whether oncogenic or tumor-suppressive programs predominate. When PTEN and RKIP are intact, coordinated restraint of PI3K/Akt and MAPK signaling biases GSK-3β activity toward growth-suppressive and differentiation-associated outcomes [172]. Conversely, simultaneous PTEN loss and RKIP downregulation shift signaling dominance toward Akt- and NF-κB-driven programs, converting GSK-3β into a facilitator of EMT, immune evasion, therapy resistance, and metastatic progression [269]. This integrated framework provides a mechanistic basis for viewing GSK-3β, RKIP, and PTEN as components of a single interconnected regulatory network rather than independent pathway modulators (Figure 9).

Together, this systems-level model substantiates the concept of functional crosstalk among GSK-3β, RKIP, and PTEN and supports the emphasis of pathway integration highlighted in the manuscript title. It also underscores the importance of assessing these regulators collectively rather than individually when interpreting tumor behavior and designing context-aware therapeutic strategies.

### Post-translational modifications as regulators of GSK-3β, PTEN, and RKIP signaling dynamics

Post-translational modifications (PTMs) play a fundamental role in regulating the activity, localization, and signaling output of GSK-3β, PTEN, and RKIP, thereby shaping pathway behavior in a highly context-dependent manner [267, 270]. Rather than functioning as static signaling components, these proteins are dynamically regulated through phosphorylation-dependent and phosphorylation-independent mechanisms that determine their tumor-suppressive or tumor-promoting functions in cancer [271].

GSK-3β activity is predominantly controlled by site-specific phosphorylation, with inhibitory phosphorylation at Ser9, mediated by Akt, p90RSK, p70S6K, and other kinases, suppressing its catalytic activity, while phosphorylation at Tyr216 is associated with kinase activation [164]. Importantly, phosphorylation does not simply switch GSK-3β “on” or “off” but can alter substrate preference, subcellular localization, and signaling integration. Consequently, the functional impact of GSK-3β phosphorylation depends on upstream pathway dominance, particularly PI3K/Akt, NF-κB, and Wnt/β-catenin signaling, providing a mechanistic basis for the observed context-dependent effects of GSK-3β inhibition in cancer [45].

PTEN is likewise subject to extensive post-translational regulation, most notably through C-terminal phosphorylation, which influences protein stability, membrane association, and phosphatase activity [272]. Phosphorylation of PTEN by kinases such as CK2 and GSK-3β can stabilize the protein but may simultaneously reduce its lipid phosphatase activity by promoting a closed conformation [273]. These modifications uncouple PTEN abundance from functional output, helping to explain why PTEN may be present but functionally impaired in certain tumors. Additional PTMs, including ubiquitination and oxidation, further regulate PTEN turnover and spatial distribution, thereby fine-tuning PI3K/Akt signaling and therapeutic responsiveness [274].

RKIP function is also strongly regulated at the post-translational level. Phosphorylation of RKIP at Ser153 by protein kinase C (PKC) induces a functional switch that releases RAF-1 and enables MAPK pathway activation while redirecting RKIP to inhibit G-protein–coupled receptor kinase-2 (GRK2) [275]. This phosphorylation-dependent target switching illustrates how RKIP can dynamically alternate between suppressing oncogenic signaling and modulating alternative pathways, depending on cellular context and upstream stimuli. Loss of unphosphorylated RKIP or accumulation of its phosphorylated form therefore has profound consequences for NF-κB activity, epithelial–mesenchymal transition, and therapy resistance [207].

Collectively, these post-translational mechanisms provide a unifying framework for understanding the dynamic and context-specific behavior of the GSK-3β/RKIP/PTEN signaling axis. PTMs enable rapid adaptation to extracellular cues, integrate multiple oncogenic pathways, and determine whether these proteins exert tumor-suppressive or tumor-promoting effects. Recognition of this regulatory layer is essential for interpreting conflicting experimental findings and for designing therapeutic strategies that account for signaling state rather than protein expression alone.

## Recent clinical and translational evidence targeting the GSK-3β/RKIP/PTEN axis

In recent years, increasing translational and early-phase clinical evidence has begun to support the therapeutic relevance of targeting the GSK-3β/RKIP/PTEN signaling network in cancer. Table 5 shows the most advanced clinical development in this area involves elraglusib (9-ING-41), a selective ATP-competitive GSK-3β inhibitor currently evaluated in multiple phase I/II studies. These trials have demonstrated acceptable safety profiles and preliminary antitumor activity, particularly when elraglusib is administered in combination with cytotoxic chemotherapy. Notably, a phase II study in metastatic pancreatic ductal adenocarcinoma reported objective responses and disease control with elraglusib combined with gemcitabine and nab-paclitaxel, supporting its role as a chemosensitizing agent rather than a standalone cytotoxic drug [276].

**TABLE 5:** Selected GSK-3β inhibitors with quantitative potency and clinical-stage information.

Compound (alias)	Mechanism of inhibition	Response rate	Adverse event frequencies	Reported GSK-3β IC_50_	Developmental/Clinical status	Notes
LY2090314 (NCT01287520) [73]	ATP- competitive	Among 35 patients, there were 5 confirmed partial responses (3 in non-small cell lung cancer, 1 in mesothelioma, and 1 in breast cancer) and 19 patients with stable disease	Eleven DLTs were reported in ten patients. Monotherapy: Grade 2 visual disturbance (n = 1) and grade 3/4 peri-infusional thoracic pain (n = 4) Combination therapy, DLTs included grade 3/4 thrombocytopenia (n = 4) and grade 4 neutropenia (n = 1)	∼0.9 nM (biochemical kinase assay)	Early-phase clinical evaluation in advanced solid tumors and hematologic malignancies	Very high biochemical potency; cellular and clinical efficacy limited by narrow therapeutic window and toxicity
9-ING-41 (elraglusib) (NCT03678883) [75]	ATP-competitive (maleimide-based)	Monotherapy (n = 62): One patient with melanoma achieved a complete response, and one with acute T-cell leukemia/lymphoma achieved a partial response Combination therapy (n = 138): Seven partial responses were observed. The median progression-free survival was 2.1 months, and the median overall survival was 6.9 months	Grade ≥3 treatment-emergent AEs occurred in 55.2% of monotherapy patients and 71.3% of combination therapy patients. Common related AEs included transient visual changes and fatigue	∼0.7 µM (biochemical assay); micromolar cellular activity	Phase I/II clinical trials (solid tumors and hematologic malignancies; monotherapy and combinations)	Lower enzymatic potency than LY2090314 but broader tolerability and combination potential

AEs: adverse events, DLTs: dose-limiting toxicities, NCT: clinical trial number, Note: IC_50_ values vary substantially depending on assay format (cell-free vs. cell-based), incubation time, and readout.

Parallel translational studies have highlighted the role of the PTEN/PI3K/Akt pathway in mediating resistance to chemotherapy and immunotherapy in clinical tumor samples. Loss of PTEN expression has been associated with poor response to immune checkpoint blockade, increased regulatory T-cell infiltration, and shortened progression-free survival, as demonstrated in recent analyses of non-small-cell lung cancer and breast cancer cohorts. These findings provide clinical support for PTEN as both a predictive biomarker and a rational target in combination treatment strategies [277].

Although RKIP remains more challenging to target directly, recent translational studies and patient-derived analyses continue to demonstrate that low RKIP expression correlates with aggressive tumor behavior, altered tumor microenvironment, and resistance to systemic therapy, reinforcing its relevance as a prognostic and functional mediator of treatment response in human cancers. Collectively, these recent clinical and translational findings underscore the importance of pathway-guided and combination-based approaches when targeting the GSK-3β/RKIP/PTEN signaling axis [278].

## Challenges in clinical translation of targeting the GSK-3β/RKIP/PTEN axis

Accumulating evidence suggests that GSK-3β, RKIP, and PTEN have potential as prognostic and predictive biomarkers in cancer; however, their clinical utility is highly context dependent and influenced by functional status and pathway activity rather than expression alone [279]. GSK-3β is not a single-direction cancer target. In some tumors it supports survival, stemness, invasion, and drug resistance, but in others, inhibition can help, do nothing, or even backfire depending on genotype and pathway state [280, 281]. The clearest failure mode is pathway rewiring. In breast cancer cell models, inhibition of GSK-3β increased resistance to doxorubicin and tamoxifen, yet the same cells became more sensitive to MEK or mTOR blockade, which says the resistance phenotype is network-dependent rather than GSK-3β-alone dependent [51]. In p53-null colon cancer, by contrast, GSK3B silencing restored chemotherapy sensitivity and drove necroptotic death, so the same target can be pro-resistance in one context and pro-death in another [282]. By another mechanism in colorectal cancer models harboring PIK3CA and TCF7 mutations, inhibition of GSK3β restored sensitivity to the dual PI3K/mTOR inhibitor gedatolisib by suppressing aberrant WNT/β-catenin signaling and reducing downstream mTOR pathway activity, thereby overcoming therapeutic resistance [283]. Collectively, these findings underscore the complexity of targeting GSK-3β as a therapeutic strategy in cancer, as its functional role is highly context-dependent and influenced by tumor genotype, signaling network rewiring, and pathway crosstalk; moreover, the majority of evidence supporting GSK-3β-targeted interventions remains largely confined to preclinical cellular and xenograft models, highlighting the need for further clinical validation before therapeutic translation.

PTEN exerts multifaceted antitumor effects through both PI3K-dependent and PI3K-independent mechanisms, while emerging evidence highlights the therapeutic potential of PTEN restoration strategies [284]. The fundamental challenge in targeting the tumor suppressor PTEN and the metastasis suppressor RKIP stems from the therapeutic necessity to restore lost function in cancers, where neither the lipid/protein phosphatase activity of PTEN nor the protein-scaffolding, kinase-inhibitory function of RKIP presents a conventional activation pocket amenable to small-molecule agonism [269, 285]. For this reason, PTEN inhibitors predominantly operate through a covalent, irreversible, and non-selective mechanism on the active-site cysteine 124 (Cys124) [286]. The design of selective, potent, and cell-permeable bivalent inhibitors is important for effectively targeting protein tyrosine phosphatases (PTPs) [287]. PTEN deficiency also correlates with immune-excluded tumor microenvironments and reduced response to PD-1/PD-L1 blockade [279]. Furthermore, the development of anticancer therapies targeting the Wnt signaling pathway has been particularly challenging because many of the most promising molecular targets are mediated through complex intracellular protein-protein interactions [288].

RKIP has primarily been investigated as a prognostic marker, where reduced expression is linked to tumor progression, metastasis, and poor survival across multiple cancers. Its predictive value remains less established due to limited clinical validation [289]. The most direct drug-discovery signal is still thin as only a few small molecules have been reported to modulate RKIP [290]. There are only eight FDA-approved compounds that increased RKIP promoter activity in breast cancer cells, but neither is a clinically validated activator [291]. The mechanistic gap is real too. RKIP downregulation remains incompletely understood in hepatocellular carcinoma despite proteasome/NF-κB-linked rescue strategies in cell lines [292]. RKIP is framed as potentially prognostic/predictive rather than a therapeutic target [293]. Despite the growing interest in RKIP as a prognostic and predictive biomarker, its clinical translation remains challenging because biomarker-based approaches, particularly those relying on immunohistochemical assessment, are often hindered by limited standardization, interstudy variability, and concerns regarding reproducibility in routine clinical practice [294]. This complexity renders RKIP a more challenging therapeutic target than single-node signaling molecules, as effective drug development requires identification of the most appropriate regulatory layer to target, consideration of tumor-specific biological contexts, and optimization of the corresponding therapeutic modality.

## Discussion

The crosstalk between GSK 3β, RKIP, and PTEN represents a tightly interconnected signaling network that regulates key oncogenic pathways, including PI3K/Akt, MAPK/ERK, Wnt/β catenin, and NF κB. Rather than acting independently, these molecules function as a coordinated axis in which PTEN suppresses PI3K/Akt signaling, RKIP inhibits Raf mediated MAPK activation, and GSK 3β integrates upstream inputs to modulate transcriptional programs. Disruption of this balance—particularly through PTEN loss and RKIP downregulation—shifts signaling toward Akt- and NF κB driven pathways, promoting proliferation, epithelial–mesenchymal transition, metastasis, and therapy resistance [3].

A key insight is the context-dependent duality of GSK 3β. While it acts as a tumor suppressor in Wnt-driven tumors by promoting β catenin degradation, it can function as a tumor promoter in cancers characterized by NF κB activation or PI3K/Akt hyperactivation. This functional plasticity is influenced by upstream signaling, cellular localization, tumor stage, and post-translational modifications such as phosphorylation at Ser9 and Tyr216 [2, 3]. These findings explain previously conflicting reports and indicate that therapeutic targeting of GSK 3β must be guided by tumor-specific signaling context rather than applied universally.

RKIP and PTEN are critical tumor suppressors that are frequently co-dysregulated and linked through regulatory circuits such as the NF-κB/Snail/YY1 loop. Hyperactivation of NF κB suppresses both RKIP and PTEN, reinforcing a feed-forward mechanism that enhances survival signaling, EMT, and resistance to therapy. Clinically, reduced expression of these molecules is consistently associated with poor prognosis, increased metastasis, and diminished response to both chemotherapy and immunotherapy [7, 231].

An important mechanistic observation is that the functional activity of this axis is not determined solely by expression levels but is dynamically regulated by post-translational modifications. Phosphorylation-dependent modulation alters GSK 3β substrate specificity, PTEN stability and activity, and RKIP functional switching, thereby shaping pathway output in a context-dependent manner [242, 244]. This provides a mechanistic basis for tumor heterogeneity and limits the usefulness of expression-based biomarkers alone.

Dysregulation of this network contributes significantly to therapeutic resistance. PTEN loss sustains PI3K/Akt signaling and promotes immune evasion, while RKIP downregulation enhances NF κB–mediated anti-apoptotic pathways. GSK 3β further modulates these effects by regulating apoptosis, EMT, and immune checkpoint expression. Notably, GSK 3β inhibition can enhance antitumor immunity and sensitize tumors to chemotherapy in selected contexts, although these effects remain highly context-dependent [268].

Translationally, emerging evidence supports targeting this axis through combination strategies. GSK 3β inhibitors such as elraglusib (9 ING 41) show early clinical promise, particularly as chemosensitizers, while PTEN status is increasingly recognized as a predictive biomarker for immunotherapy. However, challenges remain, including pathway rewiring, limited clinical validation, and lack of biomarker standardization [276].

In summary, the GSK 3β/RKIP/PTEN axis functions as a dynamic signaling hub that governs tumor progression and therapeutic response. A systems-level, context-guided approach is essential to effectively target this network and improve precision oncology outcomes.

## Future perspectives

Future research should focus on context-specific targeting of GSK-3β, given its dual and substrate-dependent roles in cancer. Rather than complete inhibition, selective modulation and activity-state–guided approaches, supported by predictive biomarkers, may better identify responsive patient subsets. Combination therapies represent another key direction, particularly integrating GSK-3β targeting with PI3K/Akt, MAPK, DNA-damage response, or immune checkpoint inhibition. Incorporating PTEN and RKIP status into trial design may improve patient stratification and treatment response.

RKIP-related strategies, including modulation of its phosphorylation state, restoration of expression, or targeting upstream regulatory loops (e.g., NF-κB/Snail), may offer indirect approaches to reverse resistance. Additionally, combining pathway-targeted agents with immunotherapy is promising, given the roles of GSK-3β and PTEN in immune regulation. Finally, emerging technologies such as single-cell profiling and spatial transcriptomics will be essential to resolve pathway heterogeneity and resistance evolution.

Multi-omics subpathway identification can reveal patient-specific dysregulated pathway regions and nominate genes implicated in oncogenic programs, enabling detection of signaling modules for further validation [295]. RKIP modulation of GSK-3β protein levels is reported in molecular analyses linking RKIP expression to changes in GSK-3β abundance, which could be captured by integrated proteogenomic profiling [296]. A modular, explainable machine learning framework for precision oncology can integrate multi-omics and multimodal tumor data to generate personalized, counterfactual treatment recommendations by aggregating specialized ensemble models, while addressing high-dimensionality and observational treatment bias and providing calibrated confidence and interpretable clinical decision support [297]. Currently, there is AttentioFuse which is an interpretable deep learning framework that integrates multi-omics data using attention-based fusion to improve cancer outcome prediction while enabling biologically meaningful interpretation and supporting the development of personalized, mechanism-guided therapeutic strategies [298].

There is a transformative role of artificial intelligence in oncology, particularly in enhancing biomarker discovery from large-scale biomedical data to improve early cancer detection, precision treatment, and clinical outcomes, while also addressing key challenges related to data quality, transparency, and ethical considerations [299]. RKIP is reported as a clinically relevant biomarker inversely associated with EMT transcription factors and linked to prognosis [296], indicating RKIP could be a candidate feature in artificial intelligence (AI) stratifiers. Future research should focus on systematically mapping synthetic lethal vulnerabilities within the GSK-3β/RKIP/PTEN signaling axis using clustered regularly interspaced short palindromic repeats (CRISPR)-based functional screens and multi-omics integration to identify compensatory dependencies and evaluate their therapeutic exploitability in combination with immunotherapy and targeted agents.

## Limitations

Despite significant progress, several limitations remain. First, the context-dependent and pleiotropic nature of GSK-3β complicates interpretation of its role across tumor types, limiting generalization of therapeutic and biomarker applications. Second, much of the current evidence is derived from *in vitro* and preclinical models, which may not fully reflect the complexity of human tumors and their microenvironment.

Third, although PTEN loss and RKIP downregulation are associated with poor prognosis and therapy resistance, their clinical utility as biomarkers is limited by methodological variability, lack of standardization, and insufficient prospective validation. Similarly, GSK-3β biomarker strategies remain underdeveloped, as activity is not reliably captured by expression levels alone.

Fourth, resistance mechanisms driven by pathway crosstalk and compensatory signaling remain incompletely understood. Finally, clinical data on GSK-3β inhibitors are still limited, with most evidence derived from early-phase trials.

Overall, further integration of systems biology, standardized biomarkers, and biomarker-driven clinical studies is required to enable effective clinical translation.

## Conclusion

In summary, the pathological consequences observed in cancer are rarely driven by dysregulation of a single molecule, but rather by disruption of the integrated GSK-3β/RKIP/PTEN signaling circuitry. The close interaction between GSK-3β, RKIP, and PTEN forms a crucial signaling network that helps control how cancer cells grow, survive, and respond to treatment. When this balance is disturbed, cancer cells gain the ability to spread and resist therapy, making these molecules central to understanding why many treatments fail. By exploring how these pathways influence one another, researchers are uncovering new possibilities for restoring normal cell behavior and improving therapeutic outcomes. Medications targeting GSK-3β, RKIP, and PTEN could open the door to more precise and durable cancer treatments. Continued research in this area may turn this complex molecular dialogue into practical, patient-centered strategies for better cancer care.
